# Branched-chain amino acids from plants and the metabolic syndrome: pathways and pharmacological applications

**DOI:** 10.3389/fnut.2026.1805807

**Published:** 2026-05-05

**Authors:** Song-nan Wang, Yu-xi Liu, Gui-yan Sun, Xin Liu, Yue Sun, Yi-xi Ma, Shun-yu Ning, Yan Shi

**Affiliations:** 1 Liaoning University of Traditional Chinese Medicine, Shenyang, China; 2 The Second Affiliated Hospital of Liaoning University of Traditional Chinese Medicine, Shenyang, China; 3 The Affiliated Hospital of Liaoning University of Traditional Chinese Medicine, Shenyang, Liaoning, China

**Keywords:** chronic inflammation, dietary protein quality, gut microbiota, insulin resistance, metabolic syndrome, mTORC1 signaling, plant-derived branched-chain amino acids, precision nutrition

## Abstract

**Background:**

Metabolic syndrome (MetS) affects approximately 1.54 billion adults worldwide and is characterized by central obesity, insulin resistance, hypertension, and dyslipidemia. Chronic low-grade inflammation is central to the development of MetS. A global shift toward a plant-based diet has prompted a reevaluation of the differing impacts of protein sources on metabolic health.

**Objective:**

The purpose of this review is to provide a systematic overview of how BCAAs from plant sources may modulate chronic inflammatory processes and improve individual components of MetS by analyzing their sources, bioavailability (including both digestibility and absorptive efficiency), metabolism, and modes of action compared to animal-based BCAAs.

**Results:**

Dietary plant-derived BCAAs are abundant and have acceptable bioaccessibility. Legumes, whole grains, and microalgae contain significant levels of BCAAs. Processing technologies such as fermentation, heat treatment, and extrusion enhance BCAA content in food products. Germination and enzymatic hydrolysis can significantly improve digestibility. Preclinical evidence from cell-based and rodent studies indicates that plant-derived BCAAs exert multi-target regulation of chronic inflammation, including modulation of mTORC1 signaling, suppression of the NF-κB pathway, potential regulation of the NLRP3 inflammasome, and beneficial interactions with gut microbiota. Their comparatively slower absorption kinetics relative to animal-derived BCAAs offer a mechanistic rationale for avoiding chronic mTORC1 hyperactivation, although direct human evidence confirming these specific mechanistic pathways remains limited and requires further clinical validation. Large prospective cohort studies suggest that substituting animal proteins with plant sources is associated with an approximately 8–12% lower risk of all-cause and cardiovascular mortality, although the certainty of this evidence is limited by residual confounding inherent in observational designs. RCT evidence (moderate certainty by GRADE) demonstrates improved glycemic control, lipid profiles, and body composition with plant-based dietary patterns, particularly in individuals with type 2 diabetes or metabolic syndrome. The “BCAA paradox” can be explained by the differing kinetics of absorption, matrix effects, and gut microbiota interactions between plant and animal sources.

**Conclusion:**

Plant-based BCAAs hold great potential for the prevention and treatment of MetS, owing to their low saturated fat content, abundant dietary fiber, polyphenolic antioxidant capacity, and beneficial effects on gut microbiota. Future studies should focus on precision nutrition, long-term clinical trials, and optimal dosing regimens. Translating mechanistic knowledge into dietary recommendations is essential for managing MetS.

## 1 Introduction

The metabolic syndrome (MetS), one of the most significant public health problems of the 21st century, is characterized by central obesity, insulin resistance, hypertension, and dyslipidemia. These conditions significantly predispose individuals to type 2 diabetes mellitus (T2DM) and cardiovascular disease (CVD) (1). According to the internationally used definition, MetS is characterized by having at least three out of several possible risk factors, including high blood pressure, hypertriglyceridemia, low levels of HDL cholesterol, elevated fasting glucose, and central obesity (1).

The global burden of MetS is increasing. The prevalence ranges from 12.5 to 31.4 per cent in a meta-analysis involving more than 28 million adults worldwide, depending on the diagnostic criteria used. The highest rates were observed in the Americas and Eastern Mediterranean regions (2). More recent data indicate a dramatic increase over the past two decades. Between 2000 and 2023, prevalence rose from 14.7 to 31.0% in women and from 9.0 to 25.7% in men. Approximately 1.54 billion adults worldwide are currently affected by it (3). The total incidence in plateau areas is as high as 30.3% (4) MetS accelerates the development of CVD and T2DM (5). New prevention methods are urgently needed.

Chronic low-grade inflammation plays a central role in the pathogenesis of MetS. In this case, there is no infection and minimal tissue damage compared to classic inflammation. The term “metainflammation” was coined for this process (6). Adipose tissue is not just inert storage but also an active endocrine organ. Several different adipokines secreted by fat tissue play roles in the inflammatory process (6).

Visceral adipose tissue expansion in obesity causes adipocyte hypertrophy, hypoxia, and cell stress, triggering the activation of the innate immune system (7). Interleukin-6 (IL-6) and tumor necrosis factor-alpha (TNF-*α*) are primarily secreted by adipocytes. The levels of these two cytokines correlate with body fat distribution and induce the synthesis of acute-phase proteins (7). Inflammatory pathways can be activated by bacterial lipopolysaccharides present in gut microbiota via pattern recognition receptors. This phenomenon is referred to as “metabolic endotoxemia” (8).

High levels of TNF-*α*, IL-6, and CRP are associated with insulin resistance, endothelial dysfunction, and atherogenesis (6, 7, 9). Insulin resistance is induced through the inhibition of IRS-1 phosphorylation by TNF-α (9). In contrast, the HMGB-1 level in MetS patients was significantly higher than that in the control group (68.5 ± 59.5 vs. 36.3 ± 48.2 ng/mL, *p* = 0.011). The level of TNF-α is also different (*p* = 0.027) (10). Chronic inflammation is both a consequence and a cause of MetS development (6).

Branched-chain amino acids (BCAAs)—leucine, isoleucine, and valine—are essential amino acids that account for approximately 35% of essential amino acids and must be obtained through diet (11). Hepatic branched-chain aminotransferase (BCAT) expression is low; therefore, dietary BCAAs enter circulation intact after meals. Blood levels directly reflect dietary intake (11, 12).

The BCAAs are substrates for the synthesis of proteins, including those in muscles; leucine potently regulates protein turnover by activating the mechanistic target of rapamycin complex 1 (mTORC1), and leucine stimulation alone is sufficient to activate mTORC1 signaling (12, 13). BCAAs are also involved in energy metabolism, and preclinical studies have established their roles as signaling molecules in the regulation of glucose homeostasis, lipid metabolism, and insulin signaling (12). The extent to which these signaling functions operate equivalently in humans under physiological dietary conditions remains an active area of investigation.

BCAA catabolism begins with the BCAT-catalyzed conversion to branched-chain *α*-keto acids (BCKAs). The rate-limiting step involves the branched-chain α-keto acid dehydrogenase complex (BCKDH), which is negatively regulated by BCKDK and positively regulated by PP2Cm (11, 12).

As discussed above, many studies have shown that plasma BCAA concentrations increase under conditions of obesity and IR. Such increases appear to be closely linked to MetS, type 2T2DM, and CVD risk (12, 14). Two mechanistic hypotheses linking elevated circulating BCAAs with T2DM have been proposed, largely based on preclinical data: first, chronic mTORC1 activation leading to uncoupling of insulin signaling via S6K1-mediated IRS-1 serine phosphorylation; second, the accumulation of potentially mitotoxic BCAA metabolites promoting *β*-cell dysfunction (12). While epidemiological data are consistent with these mechanisms, their direct causal operation in humans has not been conclusively demonstrated through interventional studies. In obesity, pro-inflammatory cytokines, lipotoxicity, decreased adiponectin, and downregulation of PPARγ disrupt BCAA metabolism, leading to excess BCAA accumulation (14). BCKAs and 3-hydroxyisobutyrate may impair insulin signaling, inhibit adipogenesis, and cause inflammation (14).

However, with the global shift toward a plant-based diet, there is an increasing interest in examining other protein sources for their impact on metabolic health (15). One meta-analysis showed that an increase in dietary BCAAs raises circulating concentrations and worsens glucose tolerance, with the largest effects observed alongside an overall increase in protein intake. The effects of BCAAs were significantly influenced by the background diet (16).

In a 16-weekRCT, 75 overweight adults received either PB diets (*n* = 38) or control diets (*n* = 37). Subjects on PB diets had <75% BCAA intake compared to controls. Lower dietary leucine intakes were strongly correlated with lower fat mass (*r* = +0.40; *p* < 0.001). Lower histidine intake was independently associated with lower insulin resistance (*r* = +0.38; *p* = 0.003). None of these associations were modified by BMI or changes in energy intake (17).

In this review, we have systematically summarized the roles that plant-based BCAAs play in MetS, particularly focusing on how they interact with metainflammation. We hope our work can provide new insights into nutritional interventions for the prevention and treatment of MetS. Human beings obtain ample proteins as well as branched-chain amino acids (BCAAs) from plant foods. The content and composition vary among different types of plants, varieties, growing conditions, and processing methods. Knowledge about the BCAA content profiles of various plant sources is important to improve plant-based dietary patterns and fulfill nutritional needs.

## 2 Sources and bioavailability of plant-derived branched-chain amino acids

Plant-based foods provide abundant protein and branched-chain amino acids (BCAAs) for humans. Their content and composition vary by plant species, variety, growing conditions, and processing methods. Understanding the BCAA content characteristics of different plant sources is essential for optimizing plant-based dietary patterns and meeting nutritional requirements. Table 1 presents the concentration profiles of branched-chain amino acids (BCAAs) in key plant-based food sources.

**Table 1:** Comparison of BCAA content in major plant-based food sources.

Food source	Total protein (g/100 g)	Leucine (mg/g protein)	Isoleucine (mg/g protein)	Valine (mg/g protein)	Key references
Legumes					
Soybean	36.5	78	46	48	(18)
Pea Protein Isolate	80–85	82	45	50	(18–20)
Lentil	25.8	76	45	51	(18)
Chickpea	19.3	73	43	44	(18)
Cereals and pseudocereals					
Quinoa	14.1	69	42	48	(21, 22)
Brown rice	7.9	82	41	59	(18)
Oats	16.9	78	45	53	(18)
Nuts and seeds					
Almond	21.2	73	41	49	(18)
Pumpkin seed	30.2	78	43	52	(18)
Microalgae					
Spirulina	57.5	88	57	62	(23, 24)
Animal-derived proteins (Reference)					
Whey protein	80–90	105	55	55	(18)
Casein	80–85	95	48	62	(18)

Data compiled from references [18–24]. Actual content may vary depending on variety, growth conditions, and processing methods. Whey protein and casein data are included as animal-derived reference values. BCAA values are expressed as mg per g of protein.

### 2.1 Major plant sources

#### 2.1.1 Legumes

Legumes are important sources of plant-based protein and BCAAs. A systematic analysis of commercially available plant-based protein isolates using UPLC–MS/MS revealed significant variation in essential amino acid content. Oat (21%), lupin (21%), and wheat (22%) had lower essential amino acid contents than animal-based proteins. Whey contained 43%, milk 39%, casein 34%, and egg 32%. Human skeletal muscle protein contains 38% (18).

Several plant-based proteins meet essential amino acid requirements, including soy (27%), brown rice (28%), pea (30%), corn (32%), and potato (37%). Leucine contents of plant proteins ranged from 5.1% for hemp to 13.5% for corn. In comparison, milk contains 9.0%, egg 7.0%, and human skeletal muscle protein 7.6% (18).

Soy, microalgae, and pea contain 4.6, 5.3, and 5.9% lysine, respectively. However, they are low in methionine. Corn, hemp, and brown rice contain 1.7, 2.0, and 2.5% methionine, respectively, but are low in lysine. Potato protein contains sufficient levels of both lysine (6.0%) and methionine (1.6%). Hemp (5.1% leucine) and lupin (5.2% leucine) do not meet the WHO/FAO/UNU requirement for leucine of 5.9%. Soy, pea, brown rice, potato, and corn provide well above the leucine requirements (18).

A double-blind randomized controlled trial (*n* = 161 males, aged 18–35, 12 weeks) compared pea protein versus whey protein supplementation on muscle thickness during resistance training. Participants were randomized into pea protein (*n* = 53), whey protein (*n* = 54), or placebo (*n* = 54) groups. They took 25 g of protein or placebo twice daily during the 12-week training period. Biceps brachii muscle thickness increased significantly over time ($*p* < 0.0001$), rising from 24.9 ± 3.8 mm at baseline to 26.9 ± 4.1 mm at day 42 and 27.3 ± 4.4 mm at day 84. Between-group differences showed only a trend toward significance ($*p* = 0.09$). In participants with lower baseline strength (1-RM < 25 kg), pea protein supplementation resulted in a + 20.2 ± 12.3% increase in muscle thickness, compared to +15.6 ± 13.5% for whey and +8.6 ± 7.3% for placebo ($*p* < 0.05$). A significant difference was found between pea and placebo. No significant difference existed between pea and whey protein groups (19).

A double-blind randomized controlled trial in healthy older males (*n* = 31, mean age 72 ± 4 years) compared the effects of whey, pea, and collagen protein supplementation on integrated myofibrillar protein synthesis (MPS) rates. Participants first underwent a 7-day control phase with protein intake fixed at the RDA level (0.8 g/kg/d). This was followed by a 7-day supplemental phase with an additional 50 g of protein daily, divided into two 25 g servings at breakfast and lunch. Compared to the RDA phase, integrated MPS rates were significantly increased in both the whey and pea protein groups. Whey showed 1.59 ± 0.11%/d versus 1.46 ± 0.09%/d (*p* < 0.001), while pea showed 1.59 ± 0.14%/d versus 1.46 ± 0.10%/d (*p* < 0.001). No change was observed with collagen supplementation. The supplemental protein provided at breakfast and lunch, above the current RDA, enhanced anabolic signaling and integrated MPS in older males. The source of additional protein may be important in overcoming age-related anabolic resistance (20).

#### 2.1.2 Cereals and pseudocereals

The lysine content of several cereals falls below WHO/FAO/UNU requirements (4.5%). Wheat contains 1.4%, corn 1.5%, oat 2.1%, brown rice 2.4%, hemp 2.8%, and lupin 3.5%. Methionine contents were low in microalgae (0.0%), oat (0.2%), lupin (0.3%), pea (0.4%), soy (0.4%), and wheat (0.9%). Potato (1.6%), corn (1.7%), hemp (2.0%), and brown rice (2.5%) met the WHO/FAO/UNU requirement (1.6%). Except for potato protein, the contents of branched-chain amino acids isoleucine and valine were lower in plant-based compared to animal-based proteins, failing to meet WHO/FAO/UNU requirements (18).

Quinoa, as a pseudocereal, is considered a complete protein. An analysis of amino acid profiles from 100 quinoa samples grown in Washington State showed that the mean essential amino acid values met WHO daily requirements for all age groups, with the only exception being the amount of leucine required by infants (21). The nutritional quality of quinoa protein has been determined through animal feeding experiments. Raw quinoa protein shows net protein utilization (NPU) of 75.7, biological value (BV) of 82.6, and true digestibility (TD) of 91.7 (22).

#### 2.1.3 Microalgae

Microalgae such as Spirulina represent unique sources of plant-based protein with exceptional nutritional profiles. Spirulina became well-known after NASA successfully used it as a dietary supplement for astronauts on space missions. It has a very high protein content, up to 60–70% dry weight, and contains vitamins, especially B12 and beta-carotene, as well as minerals, particularly iron (23). Spirulina boasts a very high content of macro- and micronutrients, including essential amino acids, proteins, lipids, vitamins, minerals, and antioxidants. It is considered a complete food supplement to combat nutritional deficiencies in developing countries (24).

### 2.2 Bioavailability of plant-derived BCAAs

In this review, bioavailability is used as a comprehensive term encompassing bioaccessibility (the fraction released from the food matrix during digestion) and true absorptive efficiency at the intestinal level, consistent with current nutritional nomenclature.

#### 2.2.1 Digestibility characteristics

**Figure 1:**
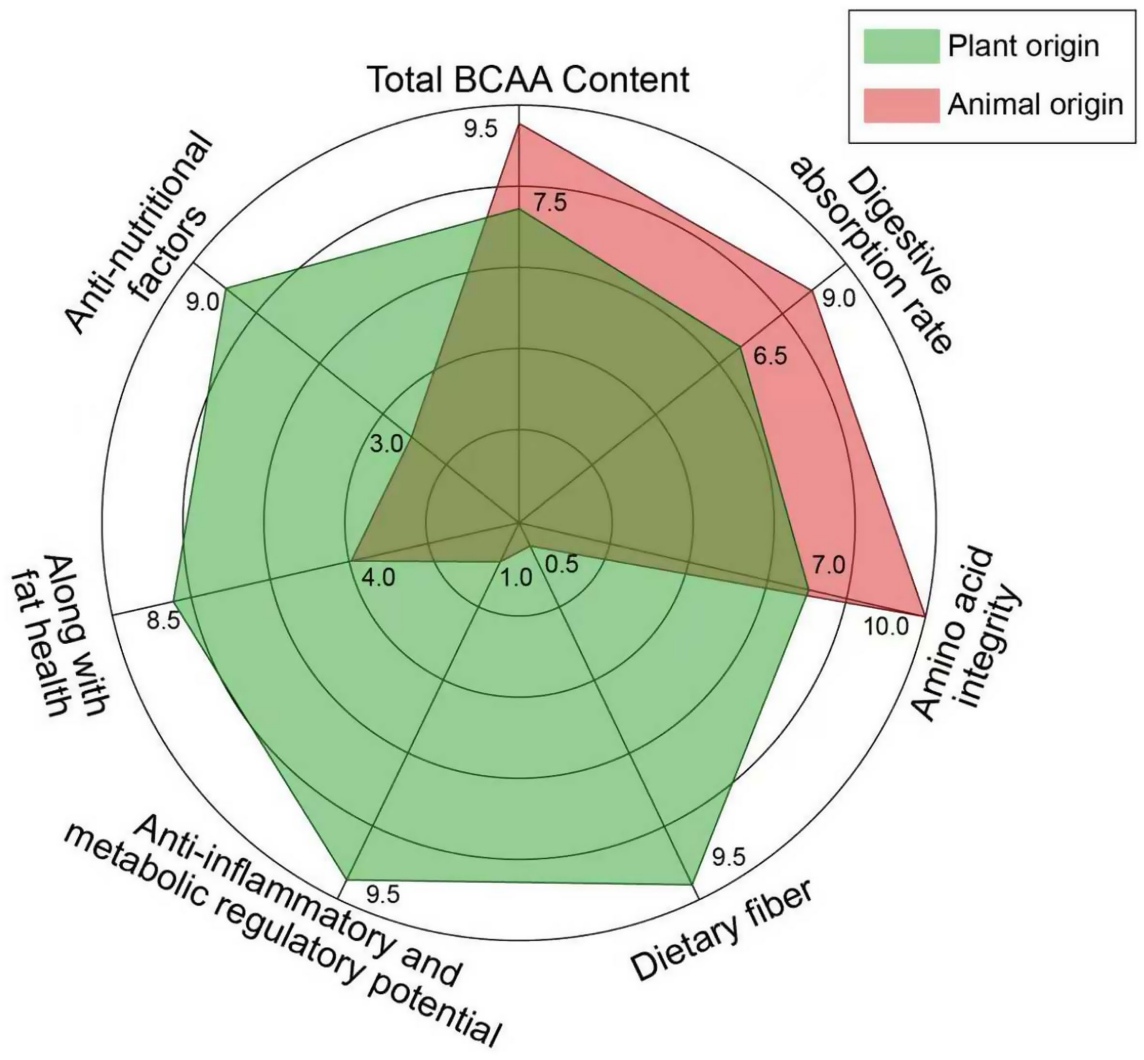
Comparative nutritional profiles of plant-derived versus animal-derived BCAA sources. Radar chart comparing the nutritional characteristics of representative plant-based (green, e.g., soy protein isolate, pea protein isolate, spirulina) versus animal-based (red, e.g., whey protein, casein, egg protein) BCAA sources across seven dimensions. Scores (0–10) represent semi-quantitative relative ratings derived from standardized values reported in the referenced literature (18, 25–27, 29, 30), normalized to the highest observed value within each dimension (= 10). The seven scoring dimensions are defined as follows: (1) *BCAA content*: Total BCAA content (leucine + isoleucine + valine) as a percentage of total protein. Animal proteins score higher (e.g., whey ~21.5% of protein); plant proteins score lower (e.g., soy ~17.2%) (18). (2) *Digestibility*: Protein digestibility expressed as true ileal digestibility (TID, %) or digestible indispensable amino acid score (DIAAS). Animal proteins typically score >90%; plant proteins range from 45 to 97% depending on processing (25, 26). (3) *Dietary fiber content*: Total dietary fiber content per 100 g of food (g). Plant sources score 8–10 (legumes: 15–25 g/100 g dry weight); animal sources score 0 (no dietary fiber) (18). (4) *Phytochemical/antioxidant capacity*: Relative antioxidant capacity and polyphenol content, scored based on DPPH/FRAP assays reported in the literature. Plant sources score 7–10; animal sources score 0–1 (29, 30). (5) *Favorable fatty acid profile*: Ratio of unsaturated to saturated fatty acids (PUFA+MUFA/SFA ratio). Higher scores reflect lower saturated fat and higher unsaturated fat content, associated with reduced cardiovascular risk. Plant sources generally score higher (e.g., soy oil PUFA: SFA ≈ 5.6); animal-derived protein foods score lower due to their associated saturated fat content (18). (6) *Anti-inflammatory activity*: Composite score based on the capacity to modulate inflammatory biomarkers (e.g., NF-κB inhibition, NLRP3 suppression, SCFA production potential from accompanying fiber), derived from *in vitro* and *in vivo* data reviewed in Section 4 (46, 55, 57). (7) *Gut microbiota modulation*: Prebiotic potential score based on the capacity of accompanying dietary fiber and polyphenols to promote beneficial microbiota (e.g., *Akkermansia muciniphila*, Bifidobacterium) and increase SCFA production. Plant sources score higher; animal sources score lower (53, 54). All scores are relative and intended for comparative illustration. Actual values vary by specific food item, variety, and processing method. Animal-based sources are superior in BCAA content and digestibility; plant-based sources excel in dietary fiber, phytochemical antioxidant capacity, favorable fatty acid profile, anti-inflammatory activity, and gut microbiota modulation.

The true ileal digestibility (TID) of four plant-based protein foods was determined in minipigs. These foods included seitan, tofu, soy milk, and pea emulsion. The TID of proteins was high and not significantly different among the foods tested. Seitan showed 97%, tofu 95%, soy milk 92%, and pea emulsion 94%. DIAAS ranking was primarily driven by the amino acid composition of the food. Soy milk scored 117%, tofu 97%, followed by pea emulsion and seitan. The lower TID of sulfur-containing amino acids in tofu compared to soy milk resulted in a significant decrease in DIAAS (from 117 to 97%). This highlights the importance of the matrix effect on nutritional protein quality (25). Figure 1 summarizes the sources and characteristics of both plant-derived and animal-derived BCAAs.

#### 2.2.2 Amino acid composition and protein quality differences

A comprehensive overview of plant and animal protein quality based on DIAAS utilized indispensable amino acid composition and standardized ileal digestibility data from 17 protein sources (5 animal, 12 plant). Based on the 0.5–3 year-old reference pattern, pork meat, casein, egg, and potato proteins are classified as excellent quality proteins (average DIAAS > 100). Whey and soy proteins are classified as high-quality proteins (average DIAAS ≥ 75). Gelatin, rapeseed, lupin, canola, corn, hemp, fava bean, oat, pea, and rice proteins are classified in the no quality claim category (DIAAS < 75). More importantly, potato, soy, and pea proteins can complement a broad range of plant proteins, leading to higher DIAAS when supplied in the form of protein mixtures at specific ratios (26).

#### 2.2.3 Impact of antinutritional factors

Antinutritional factors in plant-based foods significantly affect protein digestibility and amino acid bioavailability. Typical levels of phytates can reduce protein and amino acid digestibility by up to 10%. Tannins are present at higher levels in sorghum and certain legumes, such as fava bean, and can reduce protein and amino acid digestibility by up to 23% in rats, poultry, and pigs. High levels of dietary trypsin inhibitors from soybeans, kidney beans, or other grain legumes can cause substantial reductions in protein and amino acid digestibility, up to 50% (27).

Various traditional methods and technologies can reduce antinutritional factor levels. Legumes and cereals contain high amounts of macronutrients and micronutrients but also antinutritional factors. Major antinutritional factors include saponins, tannins, phytic acid, gossypol, lectins, protease inhibitors, amylase inhibitors, and goitrogens. Several processing techniques can be used to reduce antinutrient content in foods. These include fermentation, germination, debranning, autoclaving, and soaking. By using various methods alone or in combination, it is possible to lower the level of antinutrients in foods (28).

### 2.3 Unique accompanying components of plant-derived BCAAs

#### 2.3.1 Dietary fiber and polyphenol interactions

The nature and biological fate of polyphenol-dietary fiber conjugates from fruits and vegetables have been examined from a food and nutrition perspective. Non-extractable polyphenols bound to dietary fibers can be regenerated by extractable antioxidants in the liquid phase. The extractable antioxidants can provide electrons or hydrogen atoms to the non-extractable polyphenols, thereby regenerating them. Consequently, non-extractable ingredients with increased antioxidant capacity can be obtained. During the digestion process, they can react with free radicals while being regenerated by extractable compounds (29).

#### 2.3.2 Polyphenol–protein interactions

Two types of interactions exist between polyphenols and proteins: non-covalent interactions and covalent interactions. The latter are irreversible and more potent. The interactions between polyphenols and proteins may confer multiple benefits, including enhanced polyphenol bioavailability, promotion of protein digestion and absorption, and increased total antioxidant capacity (30).

## 3 Metabolic pathways of branched-chain amino acids

### 3.1 BCAA catabolism

**Figure 2:**
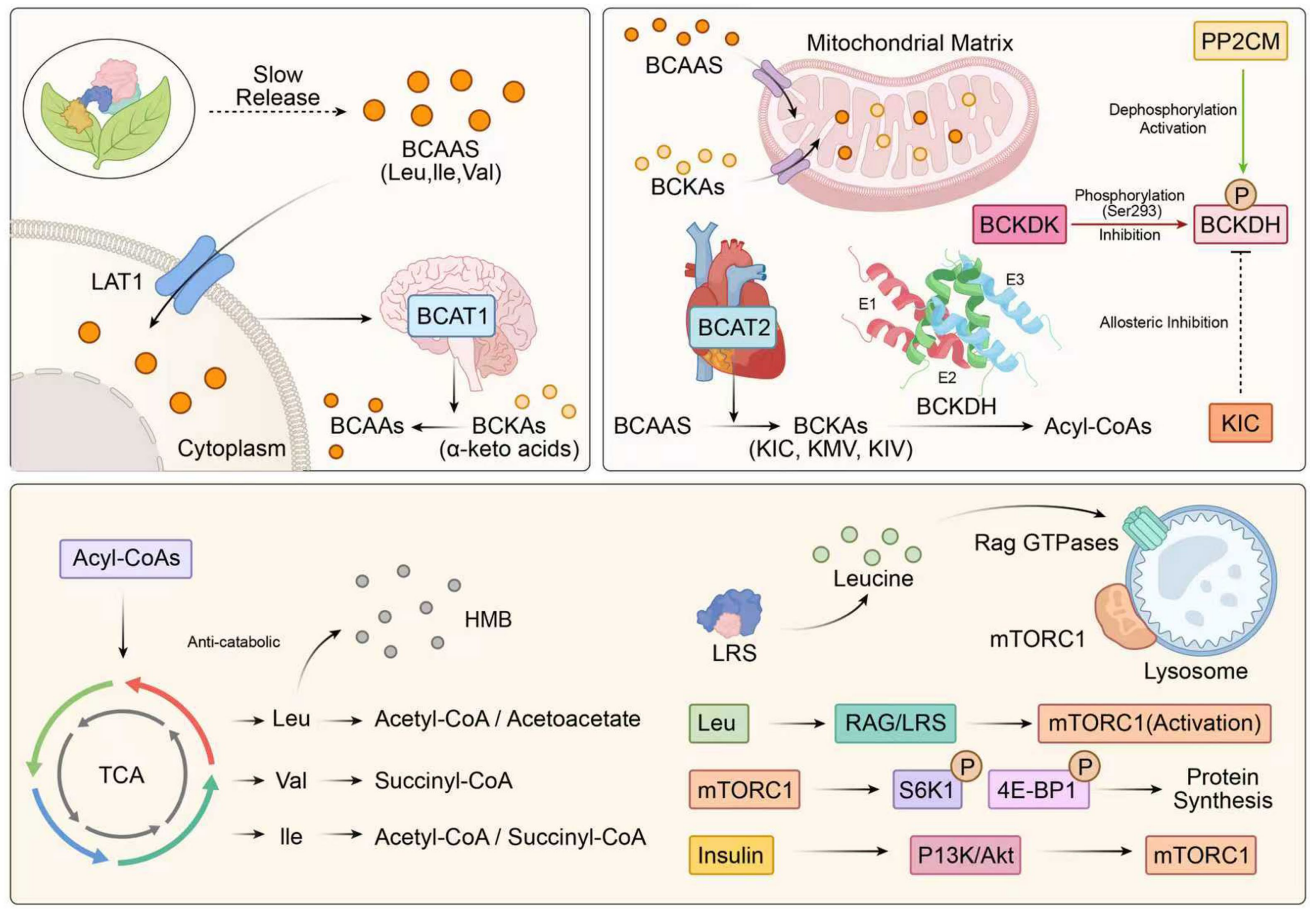
BCAA metabolic pathways and regulatory mechanisms. Schematic of BCAA transport, catabolism, and signaling. BCAAs enter the cell through the LAT1 transporter and are transaminated by BCAT1/2 into BCKAs. Catabolic flux is controlled by the mitochondrial BCKDH complex (which can be regulated by the phosphorylation of BCKDK or the dephosphorylation of PP2Cm). Leucine promotes activation of mTORC1 via LRS/Rag GTPases and synergizes with the insulin signal to stimulate protein translation by phosphorylating S6K1 and 4E-BP1. Abbreviations: LAT1, large neutral amino acid transporter 1; BCAT1/2, branched-chain aminotransferase 1/2; BCKAs, branched-chain *α*-keto acids; BCKDH, branched-chain α-keto acid dehydrogenase complex; BCKDK, BCKDH kinase; PP2Cm (PPM1K), protein phosphatase 2C family member; mTORC1, mechanistic target of rapamycin complex 1; LRS, leucyl-tRNA synthetase; Rag GTPases, Ras-related GTP-binding proteins; S6K1, ribosomal S6 kinase 1; 4E-BP1, eukaryotic translation initiation factor 4E-binding protein 1; IRS-1, insulin receptor substrate-1. Symbols: → activation/promotion; ⊣ inhibition.

BCAA catabolism is an intricate, finely tuned process that plays important roles, including maintaining nitrogen balance, energy metabolism, and metabolic homeostasis. In contrast to most other amino acids, whose major site of metabolism is the liver, the catabolism of BCAAs is specific to tissues (11). Figure 2 shows the transmembrane transport, mitochondrial catabolism, and related signaling network of BCAAs.

#### 3.1.1 Structure and regulation of the branched-chain *α*-Ketoacid dehydrogenase complex

BCAA catabolism begins with a reversible transamination reaction. Branched-chain aminotransferase (BCAT) catalyzes the conversion of leucine, isoleucine, and valine into their respective branched-chain α-keto acids (BCKAs). The BCKAs include α-ketoisocaproate (KIC), α-keto-*β*-methylvalerate (KMV), and α-ketoisovalerate (KIV) (11). A low level of mitochondrial BCAT2 isoenzyme is expressed in the liver; thus, BCAAs mostly bypass hepatic first-pass metabolism. They enter the systemic circulation and are used by peripheral tissues (11).

The rate-limiting enzyme of BCAA catabolism, branched-chain α-keto acid dehydrogenase complex (BCKDH), is tightly controlled via covalent modification. BCKDH kinase (BDK) inactivates BCKDH through phosphorylation of the E1α subunit, while BCKDH phosphatase (PPM1K) activates it through dephosphorylation (31).

In addition to regulating BCKDH, both BDK and PPM1K connect BCAA metabolism to lipid metabolism by phosphorylating another enzyme, ATP-citrate lyase (ACL) (31). Phosphoproteomic studies identified ACL as a novel substrate for both BDK and PPM1K (31). In Zucker obese rats (a preclinical rodent model of obesity), pharmacological BDK inhibition lowered circulating BCAA levels, reduced hepatic steatosis, and improved glucose tolerance (31). These findings from animal models suggest a convergence point of BCAA and lipid metabolism; however, whether analogous BDK inhibition produces comparable metabolic benefits in humans has not yet been tested in clinical trials and remains an important translational research question. This observation highlights a convergence point of BCAA and lipid metabolism, presenting new opportunities to treat metabolic disease.

#### 3.1.2 Tissue-specific metabolic characteristics

BCAA metabolism exhibits a remarkably tissue-specific distribution pattern, which is essential for understanding its physiological functions (11, 32).

##### 3.1.2.1 Skeletal muscle metabolism

Systematic *in vivo* isotopic tracing studies conducted in mice have quantified tissue-specific BCAA oxidation under healthy and insulin-resistant conditions (32). In these murine models, skeletal muscle accounts for approximately 59% of whole-body BCAA oxidation, followed by brown adipose tissue (19%), liver (8%), kidney (5%), and heart (4%) (32). While these quantitative distributions provide important mechanistic insights, it should be noted that rodent and human BCAA metabolic flux rates differ substantially, and direct isotopic tracing data from human tissues across a comparable range of metabolic conditions are currently limited. Genetic and pharmacological suppression of BDK primarily induces BCAA oxidation in the skeletal muscle of healthy mice. Insulin acutely increases BCAA oxidation in cardiac and skeletal muscle. Chronically insulin-resistant mice exhibit blunted BCAA oxidation in adipose tissues and the liver, resulting in a shift of BCAA oxidation toward muscle (32).

##### 3.1.2.2 Liver metabolism

Liver BCAT activity is relatively small, but the liver seems to play an important role during BCAA metabolism (11). Isotope tracing experiments showed that the liver is the biggest contributor to the disposal of BCAAs into proteins (approximately 27%), followed by skeletal muscle (24%) and pancreas (24%) (32). During metabolic disease states, elevated hepatic BDK expression results in higher levels of phosphorylated BCKDH with lower enzyme activity (31).

##### 3.1.2.3 Adipose tissue metabolism

Adipose tissue exerts important *in vivo* regulatory effects on BCAA metabolism (33). *In vitro* and *ex vivo* experiments suggested that adipose tissue can metabolize substantial amounts of BCAAs. However, the role of adipose tissue in regulating BCAA metabolism *in vivo* has remained controversial (33). The BCAA oxidation rate per mg of tissue was greater in adipose tissue than in skeletal muscle from wild-type mice, showing coordinated downregulation of BCAA metabolizing enzymes, which lowered the BCAA oxidation rate in adipose tissue but not in muscle, and increased circulating BCAA concentrations (33).

##### 3.1.2.4 Brown adipose tissue metabolism

Brown adipose tissue (BAT) is an active consumer of BCAAs, using them to generate heat (34). In mice, SLC25A44 has been identified as a mitochondrial transporter mediating BCAA entry into BAT mitochondria; its expression is induced by cold exposure, and knockdown of SLC25A44 suppresses BAT BCAA oxidation and attenuates BCAA-driven thermogenesis in murine models (34). These preclinical findings reveal a potentially novel role of BCAA metabolism in energy homeostasis regulation. However, the functional significance of SLC25A44-mediated BCAA catabolism in human BAT, particularly given the substantially smaller and more variable BAT mass in adult humans compared to rodents, has not been directly established.

#### 3.1.3 Metabolic intermediates and their physiological significance

Complete oxidation of BCAAs generates multiple biologically active metabolic intermediates (11).

##### 3.1.3.1 TCA cycle intermediates

Leucine, as well as isoleucine, is finally metabolized to acetyl-CoA, which may enter the TCA cycle or be used in fatty acid and ketone body synthesis. Valine forms succinyl-CoA, directly replenishing TCA cycle intermediates (11). Using isotope tracing, it was shown that up to 20% of the carbons within the TCA cycle in pancreatic tissue come from BCAAs. Thus, the pancreas has a high dependence on BCAAs as an oxidative fuel source (32).

##### 3.1.3.2 3-Hydroxyisobutyrate

As an intermediate of valine metabolism, 3-hydroxyisobutyrate (3-HIB) possesses important signaling functions (35). 3-HIB can be secreted from muscle cells and functions as a paracrine factor to regulate trans-endothelial fatty acid transport (35). Unlike other downstream intermediates of BCAA metabolism, 3-HIB is not conjugated to coenzyme A, enabling its release from mitochondria and secretion into the extracellular space. In murine cell culture and *in vivo* mouse experiments, 3-HIB was shown to activate endothelial fatty acid uptake and promote skeletal muscle lipid accumulation, contributing to insulin resistance (35). Elevated 3-HIB levels have been documented in both db/db diabetic mice and, importantly, in the skeletal muscle of human diabetic subjects (35), providing translational support for the relevance of this pathway across species. Nevertheless, whether therapeutic reduction of circulating 3-HIB in humans produces measurable improvements in insulin sensitivity remains to be tested in interventional studies, and current evidence for this pathway in humans is limited to associative data.

### 3.2 Anabolic functions of BCAAs

BCAAs are not only substrates for protein synthesis but also act as strong anabolic signals, regulating cell growth, proliferation, and metabolism by activating various signaling pathways (36).

#### 3.2.1 Mechanisms of mTOR signaling pathway activation

Mammalian target of rapamycin (mTOR) is an orchestrator of cell growth and metabolism that integrates eukaryotic cell growth and metabolism with environmental factors, such as nutrients or growth factors (36). mTOR signaling regulates many basic cellular functions, from the regulation of protein synthesis to that of autophagy. Dysregulated mTOR signaling has been linked to cancer progression, diabetes, and aging (36). Leucine is the strongest activator of mTORC1, and the mechanisms by which it activates are well characterized. There are two main leucine sensors:

##### 3.2.1.1 Sestrin2

Sestrin2 acts as a critical leucine sensor for the mTORC1 pathway (37). In amino acid-deficient conditions, Sestrin2 binds to and inhibits GATOR2. Leucine binds to Sestrin2 at an approximate dissociation constant (Kd) of 20 μM, coinciding exactly with the leucine concentration needed for half-maximal mTORC1 activity (37). The binding of leucine causes dissociation of Sestrin2 from GATOR2, releasing GATOR2 from its inhibited state, which in turn allows GATOR2 to inhibit GATOR1. This finally results in Rag GTPase activation and mTORC1 activation. The leucine binding activity of Sestrin2 is necessary for the induction of mTORC1 by leucine (37).

##### 3.2.1.2 Leucyl-tRNA synthetase

Leucyl-tRNA synthetase (LRS) is a key leucine sensor for the mTORC1 pathway (38). LRS plays a critical role in amino acid-induced mTORC1 activation by sensing intracellular leucine concentrations. LRS directly binds to Rag GTPase in an amino acid-dependent manner and functions as a GTPase-activating protein (GAP) for RagD GTPase, promoting mTORC1 activation (38). Mutations in LRS amino acid residues that affect leucine binding render the mTORC1 pathway insensitive to intracellular amino acid levels (38).

#### 3.2.2 Regulation of protein synthesis

Following mTORC1 activation, protein synthesis is promoted through the phosphorylation of downstream effector molecules (36). Major targets include *Ribosomal S6 Kinase 1*: Activation of S6K1 stimulates ribosome biogenesis and the initiation of translation on mRNA. Multiple substrates are phosphorylated by S6K1, including ribosomal protein S6 and eukaryotic initiation factor 4B, which synergistically increase translation capacity. *Eukaryotic Translation Initiation Factor 4E-Binding Protein 1*: 4E-BP1 is phosphorylated by mTORC1, releasing its inhibitory effect on eIF4E and allowing for cap-dependent translation initiation. *Autophagy inhibition*: Autophagy is inhibited by the activation of mTORC1 via the phosphorylation of ULK1, resulting in decreased protein degradation. This dual regulation of anabolism and catabolism makes BCAAs important nutritional signals for regulating protein homeostasis.

#### 3.2.3 Crosstalk with insulin signaling

BCAA metabolism and insulin signaling are known to regulate each other in a reciprocal manner (12, 39, 40). While BCAAs commonly exhibit anti-obesity properties in rodents, circulating BCAA concentrations are often elevated in obesity. Circulating BCAAs have been linked to poorer metabolic status and the subsequent development of IR or T2DM (12). One potential pathway for this is leucine-induced sustained mTORC1 activity, which may lead to premature dissociation of the insulin response. Prolonged mTORC1 activity can cause serine phosphorylation of IRS-1 and IRS-2, disrupting signaling (12).

A 12-year prospective follow-up study of 2,422 normoglycemic individuals found that 201 developed diabetes (39). Five branched-chain and aromatic amino acids (isoleucine, leucine, valine, tyrosine, and phenylalanine) were significantly associated with future diabetes risk. A combination of three amino acids predicted future diabetes, with individuals in the highest quartile having more than a fivefold higher risk (39). These results were validated in an independent prospective cohort. The BCAA-associated metabolomic signatures are among the most robust correlations to IR from reported metabolic biomarkers (40). Notably, BCAA supplementation promoted insulin resistance only under conditions of high-fat feeding, suggesting that elevated circulating levels of BCAAs are not merely markers but also contributors to metabolic dysfunction, depending on the metabolic context (40).

### 3.3 Comparison of plant-derived versus animal-derived BCAA metabolism

BCAAs from plant and animal protein sources exhibit important differences in digestion, absorption, metabolic kinetics, and physiological effects, which have significant implications for nutritional intervention strategies (41–43).

#### 3.3.1 Differences in digestive and absorptive characteristics

Digestion and release of amino acids from plant proteins differ from those in animal proteins (41). Ingestion of plant-based proteins such as soy and wheat results in lower muscle protein synthetic responses compared to several animal-based proteins (41). The potentially lower anabolic properties of plant-based protein sources may be attributed to several factors. First, plant-based sources have lower digestibility. Second, greater splanchnic extraction and subsequent urea synthesis occur with plant protein-derived amino acids compared to animal-based proteins. Third, specific essential amino acids are relatively lacking in plant-based versus animal-based proteins (41). Most plant proteins have relatively low leucine content, which may further reduce their anabolic properties (41).

About 60% of the world’s dietary protein is obtained from plants. Plant proteins tend to be less digestible, have lower leucine levels, and may lack some other EAAs like lysine or methionine (42). Protein from animal sources (e.g., dairy, meat, and fish) is reported to have over 90% digestibility, while the digestibility score of plant proteins (e.g., rice, wheat, soy, and potato) ranges from 45 to 80% (42).

#### 3.3.2 Differences in anabolic response

The lower anabolic properties of plant proteins have been confirmed in multiple studies (41–43). The ingestion of plant-based proteins has been shown to result in a lower postprandial MPS response than the same amount of animal-based protein (43). This is likely due, at least in part, to the different rates of protein digestion and AA absorption. The AA profile may also vary. Plant-based proteins generally contain fewer EAAs and may be deficient in certain AAs, including lysine or methionine (43).

#### 3.3.3 Strategies for optimizing anabolic effects of plant proteins

Despite the aforementioned differences, the lower anabolic properties of plant proteins can be compensated for through multiple strategies (41, 43): *Increased consumption*: Eating more plant-based proteins to make up for the lesser protein quality. *Using protein blends*: Utilizing specific plant protein blends to create more balanced amino acid profiles. Ingestion of 30 g of a plant-derived protein blend composed of wheat, corn, and pea proteins stimulated muscle protein synthesis in healthy young males, with responses comparable to equivalent amounts of high-quality animal-derived proteins. *Amino acid fortification*: Fortifying plant proteins with specific deficient free amino acids. *Selective breeding*: Improving plant protein amino acid profiles through selective breeding. The efficacy of these dietary strategies on postprandial muscle protein synthesis remains to be studied.

#### 3.3.4 Clinical implications and future research directions

Prior exercise or n-3 fatty acid supplementation has been shown to sensitize skeletal muscle to the anabolic properties of dietary protein. Applying these strategies when consuming plant protein-rich diets may help maintain muscle mass during aging. Given the current state of scientific knowledge, it seems that animal-derived protein can be viewed as relatively more anabolic than plant-derived protein (42). Nevertheless, clinical research needs to be conducted to assess the anabolic properties of many plant-derived proteins in human subjects, and this must occur before we can determine if a shift toward greater use of plant proteins is associated with increased dietary protein needs (43).

### 3.4 Differential metabolic roles of leucine, isoleucine, and valine in metabolic syndrome components

Although leucine, isoleucine, and valine are collectively referred to as branched-chain amino acids (BCAAs) and share common catabolic entry points, accumulating evidence demonstrates that they exert distinct and sometimes opposing roles in the pathophysiology of metabolic syndrome (MetS). A systematic comparison of their individual contributions across key MetS components is therefore warranted.

#### 3.4.1 Insulin resistance and glucose homeostasis

Leucine is the most potent activator of mTORC1 among the three BCAAs, acting through both the Sestrin2–GATOR2 axis and the leucyl-tRNA synthetase (LRS)–RagD GTPase axis (37, 38). Chronic supraphysiological leucine exposure leads to sustained mTORC1 activation, promoting inhibitory serine phosphorylation of IRS-1 via S6K1, thereby uncoupling insulin signaling and contributing to insulin resistance (12). In contrast, isoleucine has been identified as the primary driver of adverse metabolic effects among the three BCAAs. Dietary restriction of isoleucine (by 67%) in mice dramatically improved metabolic parameters, reduced fat mass, and enhanced insulin sensitivity, effects that were more pronounced than those observed with equivalent restriction of valine and were absent with leucine restriction alone (44). Furthermore, isoleucine uniquely stimulates glucose uptake in skeletal muscle independently of insulin via activation of PI3K/Akt and GLUT4 translocation (45), suggesting a dual, context-dependent role. Valine exerts comparatively modest effects on mTORC1 signaling. Its catabolic intermediate, 3-hydroxyisobutyrate (3-HIB), is a paracrine signaling molecule secreted from skeletal muscle that promotes trans-endothelial fatty acid transport, thereby driving lipid accumulation in muscle and worsening insulin resistance (35). Elevated 3-HIB levels have been documented in both diabetic mouse models and human diabetic patients (35).

#### 3.4.2 Lipid metabolism and dyslipidemia

Leucine activates sterol regulatory element-binding protein-1 (SREBP-1) via mTOR signaling, thereby promoting hepatic fatty acid synthesis and potentially contributing to dyslipidemia under conditions of metabolic excess (46). Conversely, moderate leucine intake may activate AMPK, favoring fatty acid *β*-oxidation over synthesis (47). Valine, through its metabolite 3-HIB, directly promotes endothelial fatty acid uptake and skeletal muscle lipid deposition, creating a lipotoxic environment that amplifies dyslipidemia (35). The role of isoleucine in lipid metabolism is comparatively less characterized; however, isoleucine restriction has been shown to reprogram hepatic metabolism in a sex-specific manner, enhancing ketogenesis and promoting energy expenditure through the FGF21–UCP1 pathway, thereby reducing circulating lipid burden (44).

#### 3.4.3 Blood pressure and vascular function

Leucine is the predominant BCAA mediating vascular dysfunction. Elevated plasma leucine in obese and diabetic subjects inhibits nitric oxide (NO) production in endothelial cells through activation of glutamine:fructose-6-phosphate aminotransferase (GFAT), the rate-limiting enzyme of the hexosamine biosynthesis pathway, thereby impairing endothelium-dependent vasodilation (48). Pharmacological activation of BCAA catabolism using the BCKDK inhibitor BT2 reduced systolic, diastolic, and mean arterial pressure by approximately 10 mmHg in wild-type mice, an effect attributable primarily to reduced circulating leucine and overall BCAA burden rather than to isoleucine- or valine-specific mechanisms (49). The valine-derived metabolite 3-HIB may contribute to arterial stiffness through vascular wall lipid deposition (35). The specific vascular contribution of isoleucine remains to be fully delineated.

#### 3.4.4 Muscle protein synthesis and sarcopenia prevention

Leucine is the dominant anabolic signal among the three BCAAs. It activates mTORC1 in skeletal muscle, stimulates ribosome biogenesis, and is considered the “trigger” amino acid for initiating muscle protein synthesis (MPS), with a per-meal leucine threshold of approximately 2–3 g recommended to maximally stimulate MPS in older adults (50). Isoleucine and valine primarily serve as substrates for protein synthesis rather than as independent anabolic signals, and neither can activate mTORC1 to a comparable extent as leucine in isolation (12). However, the presence of all three BCAAs is required to sustain MPS, as isoleucine and valine are essential for the aminoacylation reactions that underpin translational elongation.

The differential roles of the three BCAAs across MetS components are systematically summarized in Table 2.

**Table 2:** Differential metabolic roles of leucine, isoleucine, and valine in components of metabolic syndrome.

MetS component	Leucine	Isoleucine	Valine
Insulin resistance	Chronic mTORC1 → S6K1 → IRS-1 serine phosphorylation promotes IR at excess (12)	Primary adverse driver; restriction reverses IR; also stimulates GLUT4-mediated glucose uptake (44, 45)	3-HIB promotes muscle lipid accumulation → lipotoxic IR (35)
Lipid metabolism	↑SREBP-1 (lipogenesis) via mTOR; moderate intake ↑AMPK (*β*-oxidation) (46, 47)	Restriction: ↑hepatic ketogenesis, ↑energy expenditure via FGF21-UCP1 (44)	3-HIB drives trans-endothelial FA transport → skeletal muscle lipid deposition (35)
Blood pressure	Inhibits NO synthesis via GFAT activation in endothelial cells (48)	Role not well characterized	3-HIB → vascular lipid deposition → arterial stiffness (35)
Muscle protein synthesis	Primary anabolic trigger; activates mTORC1; threshold ~2–3 g/meal (50)	Substrate for protein synthesis; minor mTORC1 activation (12)	Substrate for protein synthesis; minor mTORC1 activation (12)
Pancreatic *β*-cell	Most potent insulin secretagogue via GDH and mTORC1 (82)	Stimulates GLP-1 release from L-cells (7)	Modest insulin secretory effect (45).

IR, insulin resistance; mTORC1, mechanistic target of rapamycin complex 1; IRS-1, insulin receptor substrate-1; S6K1, ribosomal S6 kinase 1; 3-HIB, 3-hydroxyisobutyrate; GLUT4, glucose transporter type 4; SREBP-1, sterol regulatory element-binding protein 1; AMPK, AMP-activated protein kinase; FGF21, fibroblast growth factor 21; UCP1, uncoupling protein 1; GFAT, glutamine:fructose-6-phosphate aminotransferase; NO, nitric oxide; FA, fatty acid; GDH, glutamate dehydrogenase; GLP-1, glucagon-like peptide-1.

## 4 Mechanisms of plant-derived BCAAs in modulating chronic inflammation

**Figure 3:**
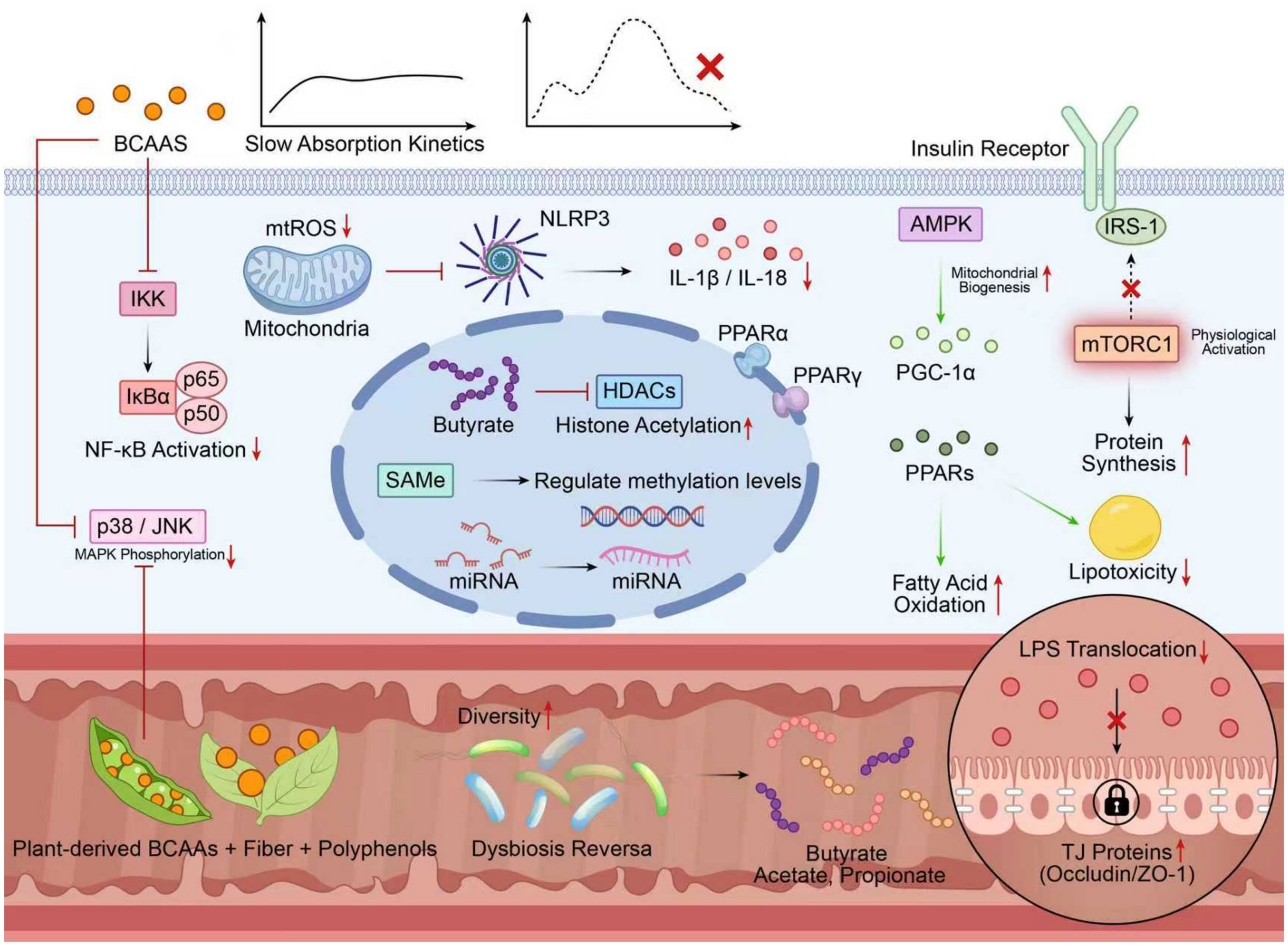
Anti-inflammatory mechanisms of plant-derived BCAAs through the gut-cell axis. Plant-derived BCAAs, along with fiber and polyphenols, modulate gut microbiota, increasing SCFAs and strengthening the intestinal barrier (through Occludin/ZO-1); slow the kinetics of absorption to prevent plasma spikes; and, synergizing with polyphenols, inhibit inflammation via suppressing NF-κB, MAPK, and NLRP3 pathways. Metabolically, they activate mTORC1 without compromising insulin sensitivity and promote mitochondrial biogenesis through AMPK-PGC-1α, as well as promote epigenetic anti-inflammatory reprogramming by inhibiting HDACs via butyrate. Abbreviations: SCFAs, short-chain fatty acids; ZO-1, zonula occludens-1; NF-κB, nuclear factor-κB; MAPK, mitogen-activated protein kinase; NLRP3, NOD-like receptor thermal protein domain-associated protein 3; AMPK, AMP-activated protein kinase; PGC-1α, peroxisome proliferator-activated receptor gamma coactivator 1-alpha; HDACs, histone deacetylases. Symbols: → activation/promotion; ⊣ inhibition.

Chronic low-grade inflammation is the common pathological basis of metabolic disease, cardiovascular disease, and various other chronic conditions. Social, environmental, and lifestyle factors may contribute to systemic chronic inflammation (SCI). This, in turn, predisposes individuals to diseases that collectively constitute the main causes of disability and death worldwide. These include cardiovascular disease, cancer, diabetes mellitus, CKD, NASH, and autoimmune as well as degenerative diseases of the nervous system (51). Branched-chain amino acids (BCAAs), along with other phytochemicals from plant sources, possess distinctive properties to modulate chronic inflammatory conditions. Figure 3 illustrates the direct and indirect ways that plant-derived BCAAs can modify chronic inflammation (i.e., anti-inflammatory effects of these amino acids through the gut-cell axis, gut microbiota, and epigenetic pathways).

### 4.1 Direct anti-inflammatory mechanisms

#### 4.1.1 Inhibition of the NF-κB signaling pathway

The transcription factor nuclear factor-κB (NF-κB) controls several aspects of both innate and adaptive immunity. It is an important mediator of inflammation, inducing the expression of various inflammatory genes, including those coding for cytokines and chemokines. It is also involved in inflammasome control, with deregulated NF-κB activation contributing to the pathogenesis of many inflammatory diseases (52).

*In vitro* studies using RAW264.7 murine macrophages demonstrated that peptides derived from soybean proteins can inhibit LPS-induced inflammation through the suppression of TLR4-mediated MAPK-JNK phosphorylation and NF-κB activation, resulting in reduced mRNA expression and protein secretion of TNF-*α*, IL-6, and IL-1β (53). These cell-based findings establish a mechanistic basis for the potential anti-inflammatory activity of soybean-derived peptides; however, it must be noted that RAW264.7 cells are a murine macrophage cell line, and whether equivalent peptide concentrations are achievable in human systemic circulation following dietary soybean protein ingestion, as well as whether analogous anti-inflammatory effects occur *in vivo* in humans, has not been directly demonstrated.

Translational note: The NF-κB inhibitory effects of plant protein-derived peptides described in this section are based exclusively on *in vitro* cell culture data. No published RCT has directly measured NF-κB pathway activity as a primary outcome following plant-derived BCAA or plant protein dietary intervention in humans. The mechanistic plausibility established by these preclinical studies should not be interpreted as clinical validation of anti-inflammatory efficacy.

#### 4.1.2 Regulation of the NLRP3 Inflammasome

The NLRP3 inflammasome plays an important role in the innate immune response. The NLRP3 (NOD-, LRR-, and pyrin domain-containing protein 3) serves as an intracellular receptor that senses a wide variety of microbial patterns, endogenous danger signals, and environmental irritants. This triggers the formation and activation of the NLRP3 inflammasome. The formation of the NLRP3 inflammasome leads to the caspase-1-dependent release of the pro-inflammatory cytokines IL-1β and interleukin-18 (IL-18), also resulting in gasdermin D-mediated pyroptotic cell death (54).

The mechanistic target of rapamycin (mTOR) couples eukaryotic cell growth and metabolism to the environment, incorporating signals from nutrients and growth factors. Deregulated mTOR signaling has been implicated in the progression of cancer and diabetes, as well as aging (36). Based on preclinical evidence, plant-based BCAAs may act as upstream modulators of mTOR signaling, potentially contributing to metabolic homeostasis through their comparatively slower postprandial absorption kinetics relative to animal-derived BCAAs. However, it must be explicitly stated that no direct experimental evidence—whether in cell culture, animal models, or human studies—has established a specific causal link between plant-derived BCAAs and NLRP3 inflammasome regulation. This proposed connection currently represents mechanistic speculation informed by pathway-level reasoning and should not be cited as established evidence. Future studies directly measuring NLRP3 activation in response to plant-derived BCAA interventions are needed.

#### 4.1.3 Nrf2-mediated anti-inflammatory effects

The transcription factor Nrf2 is a well-characterized regulator of the oxidative stress response. Cell-based studies using ChIP-seq and ChIP-qPCR have demonstrated that Nrf2 can directly inhibit the transcriptional upregulation of LPS-induced pro-inflammatory cytokines, including IL-6 and IL-1β, by binding to their proximal promoter regions and inhibiting RNA polymerase II recruitment, independently of antioxidant gene regulation (55). While these mechanistic findings are derived from *in vitro* experimental systems, Nrf2 activation by plant-derived phytochemicals (e.g., sulforaphane from cruciferous vegetables) has been partially validated in human pharmacokinetic studies (56). Nevertheless, direct evidence that dietary plant-derived BCAAs specifically activate Nrf2-mediated anti-inflammatory pathways in human metabolic tissues remains to be established. It should be noted that Nrf2’s inhibition of inflammation is independent of the regulation of antioxidant genes or intracellular ROS levels (55).

### 4.2 Indirect anti-inflammatory mechanisms

#### 4.2.1 Amelioration of metabolic inflammation

Metabolic inflammation, or “metaflammation,” is considered a central process linking the immune system and metabolism (57). Metaflammation is defined as chronic low-grade inflammation caused by a multitude of pro-inflammatory mediators. There are evolutionarily conserved interactions between immunity and metabolism. The equilibrium between the two systems must be maintained to ensure good health, while its disruption could induce chronic non-communicable diseases such as obesity or diabetes (58). It has been hypothesized that the comparatively slower postprandial absorption kinetics of plant-derived BCAAs—attributable to the food matrix effects of dietary fiber and antinutritional factors—may generate more gradual and sustained postprandial amino acid signals, theoretically avoiding the acute mTORC1 hyperactivation associated with rapid BCAA absorption from animal-derived proteins. This hypothesis is mechanistically plausible and supported by indirect evidence from pharmacokinetic comparisons of plant and animal protein digestion (41, 43); however, it has not been directly tested in controlled human studies measuring real-time mTORC1 activity or insulin signaling in response to plant versus animal BCAA sources at matched doses. This remains an important and currently unvalidated hypothesis.

#### 4.2.2 Regulation of energy metabolism

AMP-activated protein kinase (AMPK) is a well-established cellular energy sensor whose biology has been extensively characterized in cell and animal studies (59). Preclinical evidence demonstrates that AMPK activation promotes catabolic ATP-generating pathways and suppresses energy-consuming biosynthetic processes, with systemic effects on metabolic homeostasis, including circadian rhythm entrainment and hypothalamic regulation of food intake (59). While AMPK activation by various dietary components has been studied in humans (e.g., metformin, which partially activates AMPK, is a validated T2DM therapeutic), direct evidence that dietary plant-derived BCAAs specifically activate AMPK in human metabolic tissues at physiologically relevant concentrations achieved through normal dietary intake has not been established in clinical intervention studies. Additionally, AMPK regulates metabolic energy balance on a body-wide scale—for example, it transmits the effects of agents acting in the hypothalamus that stimulate eating. It also entrains circadian rhythms of metabolism and feeding behavior (59).

#### 4.2.3 Metabolic flexibility

The term metabolic flexibility describes the capacity to respond to or adapt to conditional changes in metabolic demand. Metabolic flexibility has been extensively applied to describe insulin resistance and mechanisms controlling the selection of fuels from either glucose or fat. It also highlights the metabolic inflexibility associated with obesity and T2DM (60). The potential role of plant-derived BCAAs in preserving metabolic flexibility is currently entirely theoretical and is not supported by direct human interventional evidence. This represents a mechanistic hypothesis informed by pathway-level reasoning from preclinical studies; therefore, the phrase “confirm these findings” is not appropriate in this context—there are no human findings to confirm. Future prospective clinical trials directly measuring substrate oxidation flexibility (e.g., via respiratory quotient under controlled metabolic conditions) in response to plant versus animal BCAA dietary interventions are needed before any conclusions regarding plant-derived BCAAs and metabolic flexibility can be drawn in humans.

### 4.3 Gut microbiota-mediated anti-inflammatory effects

#### 4.3.1 Modulation of microbiota structure

Diet is a key factor influencing gut microbiota composition. Short-term consumption of diets composed entirely of animal or plant products can alter microbial community structure (61). In a controlled human dietary crossover study (*n* = 10 healthy individuals), David et al. demonstrated that short-term consumption of entirely animal-based diets increased the abundance of bile-tolerant microorganisms (Alistipes, Bilophila, and Bacteroides) and decreased the levels of plant polysaccharide-fermenting Firmicutes (Roseburia, *Eubacterium rectale*, and *Ruminococcus bromii*) compared to plant-based diets (61). These direct human data represent relatively robust evidence for rapid diet-induced gut microbiota remodeling; however, the study involved extreme dietary conditions (exclusively animal or plant diets) over only 5 days, and the metabolic consequences of these microbiota shifts were not measured as primary outcomes.

In a randomized, double-blind, placebo-controlled trial, it was shown that supplementation of *Akkermansia muciniphila* is safe and well-tolerated (62). Compared with placebo, pasteurized *A. muciniphila* significantly enhanced insulin sensitivity: it increased by 28.62 ± 7.02% (*p* = 0.002), reduced insulinemia by −34.08 ± 7.12% (*p* = 0.006), and lowered plasma total cholesterol by 8.68 ± 2.38% (*p* = 0.02) in overweight and obese subjects. Following 3 months of supplementation, *A. muciniphila* also lowered blood levels of indicators of liver dysfunction and inflammation (62).

#### 4.3.2 Immunomodulatory effects of short-chain fatty acids

Short-chain fatty acids (SCFAs) are the major metabolic products of gut microbiota fermentation of dietary fiber. Among these, butyrate possesses important immunomodulatory functions. Butyrate regulates intestinal macrophage function through histone deacetylase (HDAC) inhibition mechanisms (63). Under LPS-stimulated conditions, butyrate treatment downregulated the expression of pro-inflammatory mediators, including nitric oxide, IL-6, and IL-12. However, it did not affect TNF-*α* or monocyte chemoattractant protein-1 (MCP-1) levels. This effect was independent of Toll-like receptor signaling and G protein-coupled receptor activation. Multiple lines of evidence indicate that these effects of butyrate derive from its HDAC inhibitory activity. This renders lamina propria macrophages hyporesponsive to commensal bacteria and contributes to intestinal immune tolerance (63).

Butyrate, derived from commensal microbes, induces the differentiation of regulatory T (Treg) cells in the colon (64). Using metabolomic analysis, we found that luminal levels of short-chain fatty acids were positively correlated with the number of Treg cells present in the colon. We demonstrated that butyrate could induce Treg cell differentiation *in vitro* and *in vivo*. Butyrate ameliorated the development of colitis induced by the adoptive transfer of CD^4+^CD45RBh i T cells. Mechanistic analyses showed that treatment with butyrate during Treg cell polarization increased histone H3 acetylation at the promoter region and conserved non-coding sequences (CNS) region of the *Foxp3* locus, providing a potential explanation for how microbially derived butyrate can regulate Treg cell development (64).

#### 4.3.3 Interactions between gut microbiota and dietary lipids

Dietary interactions with the gut microbiota may also affect systemic levels of metabolic inflammation. Diets high in lard, which is rich in saturated fats, have been compared to those rich in polyunsaturated fatty acids from fish oils (65). The latter diet resulted in increased TLR activation, WAT inflammation, and decreased insulin sensitivity compared to lard-fed animals. Phenotypic differences could be partially explained by changes in the composition of the microbiota. The effect of gut microbiota on SFA-induced WAT inflammation is not dependent on fat mass in an animal model using germ-free mice. The chemokine CCL2 has also been implicated in microbiota-mediated WAT inflammation in lard-fed mice: Trif−−/−− and Myd88−−/−− mice were protected from lard-induced WAT inflammation and defective insulin sensitivity (65).

These studies suggest that plant-based diets may be effective in decreasing systemic metabolic inflammation through modulation of gut microbiota composition.

## 5 Effects of plant-derived BCAAs on components of metabolic syndrome

### 5.1 Obesity and weight management

**Figure 4:**
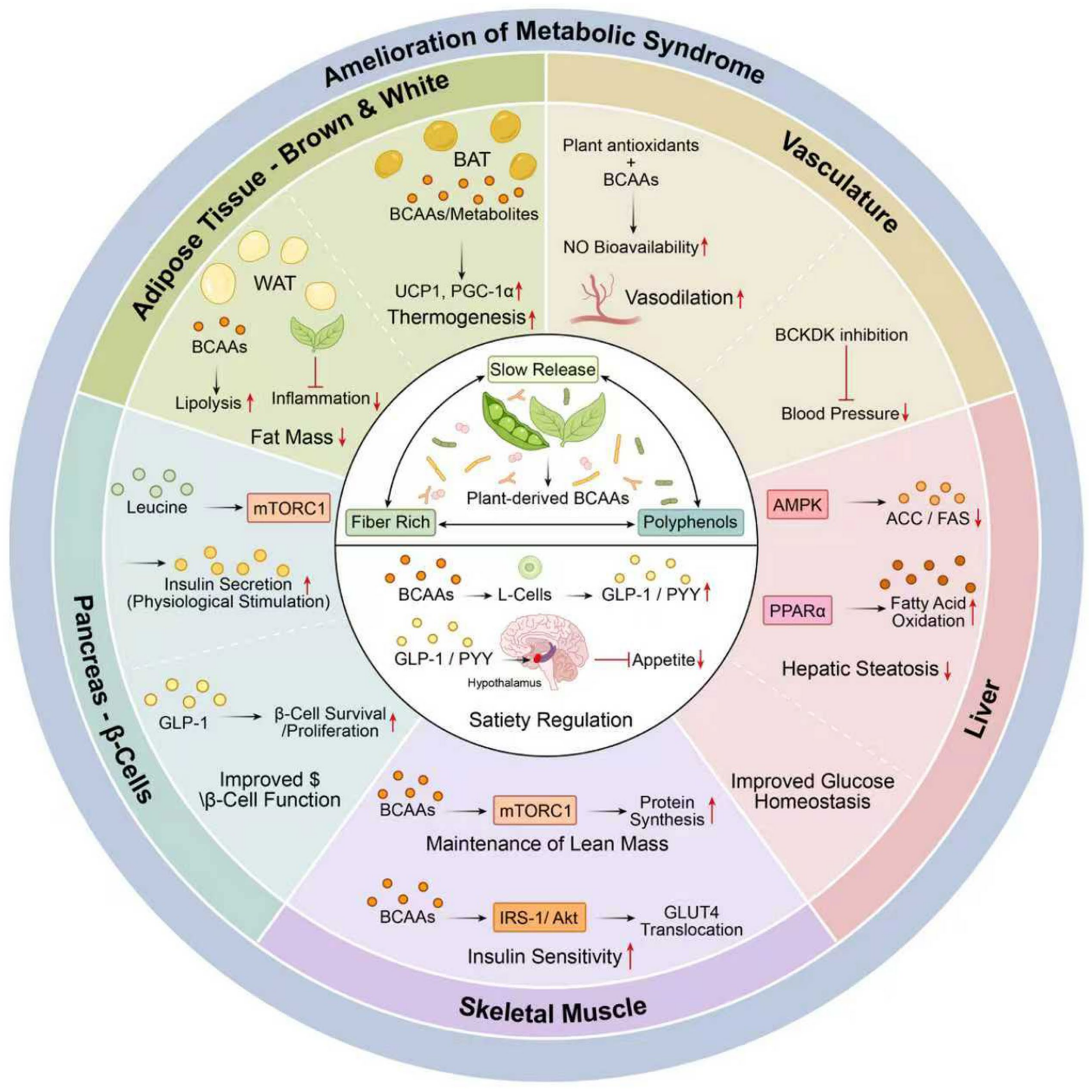
Multi-organ mechanisms of plant-derived BCAAs in improving metabolic syndrome. Plant-derived BCAAs, with slow-release kinetics, promote the secretion of GLP-1/PYY to regulate hunger and have multi-organ effects: they promote BAT thermogenesis and WAT lipolysis, improve beta cell functions of the pancreas, maintain muscle mass by mTORC1, decrease fatty liver (hepatic steatosis) by AMPK/PPARα, and improve vascular function by increasing the bioavailability of NO. All of these cooperative actions contribute to the improvement of metabolic syndrome. Abbreviations: GLP-1, glucagon-like peptide-1; PYY, peptide YY; BAT, brown adipose tissue; WAT, white adipose tissue; UCP1, uncoupling protein 1; PPARα, peroxisome proliferator-activated receptor alpha; NO, nitric oxide; mTORC1, mechanistic target of rapamycin complex 1; AMPK, AMP-activated protein kinase. Symbols: → activation/promotion; ⊣ inhibition.

Metabolic syndrome is defined as a cluster of signs and symptoms that includes central (abdominal) obesity, insulin resistance, high blood pressure, and dyslipidemia. Branched-chain amino acids (BCAAs) consist of three types: leucine, isoleucine, and valine. These amino acids are essential nutrients required to maintain physiological functions and metabolic homeostasis (14). Recent evidence has indicated that BCAAs play important roles in regulating many aspects of metabolic syndrome. Figure 4 illustrates the role of diet-induced BCAA production by plants (alongside dietary fiber and polyphenols) in improving whole-body metabolism via a hub-and-spoke system that reaches all other organ systems.

#### 5.1.1 Energy metabolism regulation

BCAAs play key roles in the regulation of energy metabolism. In murine models, dietary leucine deprivation has been shown to increase whole-body oxygen consumption and upregulate uncoupling protein 1 (UCP1) expression in brown adipose tissue, accompanied by increased PGC-1α and PPARγ expression (66, 67). These animal studies establish a mechanistic link between leucine availability and thermogenic regulation. However, it should be noted that these findings were obtained under conditions of severe leucine deprivation in mice, which is not equivalent to the moderate reductions in leucine intake associated with replacing animal protein with plant protein in human diets. Whether moderate reductions in dietary leucine through plant-based eating patterns produce clinically meaningful changes in human BAT thermogenesis or energy expenditure has not been demonstrated.

UCP1 is one of the major thermogenic proteins expressed in the mitochondria of brown adipocytes, uncoupling the proton gradient across the inner mitochondrial membrane to generate heat rather than ATP (67). PGC-1α and PPARγ are important transcription factors that regulate the expression of UCP1 (66). They promote the thermogenic function of brown fat.

At the mechanistic level, preclinical studies demonstrate that BCAAs modulate the balance between fatty acid synthesis and oxidation via mTORC1 activation (68). Human observational data indicate that reduced expression of BCAA catabolic enzymes in white adipose tissue is associated with obesity and insulin resistance (69), suggesting the translational relevance of this pathway; however, whether correcting adipose BCAA catabolism through dietary plant protein substitution causally improves this enzyme expression profile in humans has not been tested in controlled intervention studies.

#### 5.1.2 Appetite and satiety regulation

The role of BCAAs in appetite regulation has been well established. Leucine, a BCAA, serves as a nutritional signal in the hypothalamus. The reduction of food intake via leucine involves activation of mTOR. mTOR signaling in the arcuate nucleus of the hypothalamus controls energy balance. Central infusion of leucine activates mTOR signaling in the hypothalamus, leading to reduced food intake and body weight (70). The regulation of gut hormones by BCAAs is another significant mechanism for appetite control. *In vitro* studies using NCI-H716 human intestinal enteroendocrine cell lines demonstrated that leucine and isoleucine can stimulate GLP-1 secretion and decrease expression of genes related to fatty acid synthesis and absorption (71). While these cell-based findings are mechanistically informative, NCI-H716 cells represent a simplified model of intestinal L-cell physiology. Human *in vivo* studies measuring GLP-1 responses to plant-derived BCAA ingestion under controlled conditions, while distinguishing BCAA-specific effects from those of co-ingested dietary fiber and polyphenols, are needed to validate these *in vitro* observations. Clinical research has shown that plasma PYY increases and satiety scores improve when consuming wheat biscuits supplemented with BCAAs (72). Whey protein, which contains a high BCAA content, exhibits significant appetite-suppressing effects (73). Whey protein intake in obese subjects significantly elevates plasma GLP-1 and PYY levels, accompanied by increases in leucine, isoleucine, and valine concentrations (74). Eight specific amino acids (isoleucine, leucine, lysine, methionine, phenylalanine, proline, tyrosine, and valine) are negatively correlated with hunger and positively correlated with satiety and GLP-1 levels.

#### 5.1.3 Body composition improvement

BCAAs play important roles in maintaining lean body mass and improving body composition, particularly in elderly populations. Muscle protein synthesis requires all essential amino acids, and BCAAs account for approximately 50% of essential amino acids in muscle protein (75). BCAAs promote protein synthesis through activation of the mTORC1 signaling pathway and reduce protein degradation by inhibiting the ubiquitin-proteasome pathway (76).

BCAAs support lean body mass during caloric restriction. In a randomized controlled study, participants supplementing with BCAAs along with resistance exercise were able to maintain lean mass and reduce fat mass compared to controls, who experienced lean mass loss (77). A meta-analysis compared the effects of plant and animal proteins on muscle mass and showed no significant difference between soy protein and milk protein (SMD = −0.02; *p* = 0.80). This indicates that certain plant proteins can have the same effect as animal proteins in suitable circumstances (78). However, overall lean body mass was slightly lower in the plant protein group than in the animal protein group (SMD = −0.20; *p* = 0.02). This suggests that plant proteins need to be carefully selected and combined if they are to contribute to healthy muscles. For older adults, adequate intake of BCAAs to preserve lean body mass is essential for preventing sarcopenia and improving metabolic profiles. About 2-3 g of leucine per meal could effectively stimulate MPS (50). Rich plant sources include oats, lentils, and legumes, which can provide sufficient BCAA support for vegetarians.

#### 5.1.4 Clinical evidence

Clinical studies assessing the effect of BCAAs on bodyweight management yielded inconclusive results. One RCT enrolled 132 obese and/or overweight individuals. After 16 weeks of energy restriction, weight loss was similar among the normal protein, BCAA supplementation, and high protein groups (~7.97%), with the BCAA group tending to lose less lean mass (79). In another RCT involving overweight and obese women, BCAAs supplemented with vitamin B6 were associated with the preservation of leg lean mass and an improvement in the waist-to-hip ratio (80).

A meta-analysis of rodent studies demonstrated that elevated dietary BCAA levels are associated with increased circulating BCAA concentrations, a relationship that is significantly influenced by background diet (16). Impaired glucose tolerance was linked to elevated dietary BCAAs, with the effect being most pronounced when total protein intake was simultaneously increased. Dose–response experiments have suggested that the metabolic effect of BCAAs closely relates to intake quantity, duration, and the individual’s metabolic state. In metabolically healthy subjects, moderate BCAA consumption might be beneficial; however, in individuals already suffering from metabolic dysfunction, excess BCAAs could exacerbate the development of IR (81). Thus, individually tailored strategies for BCAA use are necessary.

The clinical evidence regarding BCAAs and weight management is notably heterogeneous and context-dependent. The divergent findings between Ooi et al. (2021) (82), in which BCAA supplementation during energy restriction showed only a trend toward lean mass preservation without statistical significance, and Novin et al. (2018) (80) which reported significant preservation of leg lean mass with BCAA plus vitamin B6 co-supplementation, likely reflect differences in the presence of a vitamin co-factor, the sex composition of study populations (exclusively female in Novin et al.), the degree of energy restriction imposed, and the background protein intake. Neither study adequately characterized participants’ baseline metabolic status or gut microbiota composition, both of which may substantially modify the response to BCAA supplementation. The moderate and often statistically non-significant effects observed across weight management RCTs also suggest that BCAA supplementation alone is unlikely to produce clinically meaningful weight loss or lean mass accretion without a structured exercise program and overall dietary quality improvement.

### 5.2 Insulin resistance and type 2 diabetes

#### 5.2.1 Insulin signaling modulation

A complex bidirectional regulatory relationship exists between BCAAs and insulin signaling pathways. On one hand, BCAAs can promote insulin signal transduction through mTORC1 activation. On the other hand, excessive BCAAs may increase serine phosphorylation of insulin receptor substrate-1 (IRS-1), inhibiting insulin signaling (83). In obesity and insulin resistance states, persistently elevated BCAA concentrations activate S6 kinase 1 (S6K1) downstream of mTORC1. S6K1 mediates the inhibitory serine phosphorylation of IRS-1/2, leading to insulin signaling uncoupling (11). In experimental studies using cell culture and mouse models, 3-hydroxyisobutyrate (3-HIB), a valine-derived catabolic intermediate, was shown to be secreted from skeletal muscle and to activate trans-endothelial fatty acid transport, thereby promoting intramuscular lipid accumulation and insulin resistance (35). Elevated 3-HIB has been documented in human skeletal muscle in association with diabetes (35), providing some translational support; however, whether dietary modification—specifically increasing plant protein intake—reduces circulating 3-HIB and improves insulin sensitivity through this specific mechanism in humans has not been demonstrated in clinical intervention studies. 3-HIB is an intermediate product of valine catabolism, and its levels are significantly elevated in the muscle tissue of both diabetic mouse models and human diabetic patients. Dietary BCAA restriction in Zucker fatty rats improves skeletal muscle insulin sensitivity (84). BCAA restriction completely normalizes the accumulation of fatty acyl-CoAs in skeletal muscle and improves metabolism by increasing fatty acid oxidation efficiency as well as excretion of acyl-glycines. Thus, targeting BCAA metabolism may be a promising approach to improving insulin signaling.

#### 5.2.2 Pancreatic *β*-cell function

Preclinical studies suggest that BCAAs exert concentration-dependent dual regulatory effects on pancreatic *β*-cell function: at physiological concentrations, BCAAs—particularly leucine—stimulate insulin secretion through activation of glutamate dehydrogenase and mTORC1 signaling in β-cell culture models and rodent studies (82). In contrast, chronic supraphysiological BCAA exposure in animal models has been associated with the accumulation of potentially mitotoxic metabolites (branched-chain *α*-keto acids and acylcarnitines), contributing to β-cell mitochondrial dysfunction and apoptosis (83). Translational evidence in humans is limited: epidemiological data show associations between elevated circulating BCAAs and impaired β-cell function in cross-sectional studies, but whether the proposed mitotoxic mechanism operates in human β-cells under dietary conditions achievable through normal food intake has not been directly demonstrated in interventional studies. In T2D patients, β-cells do not respond appropriately to BCAAs; insufficient insulin is secreted. Synergistic effects exist between BCAAs and GLP-1. Leucine and isoleucine stimulate GLP-1 release from intestinal L cells (7). This not only enhances glucose-dependent insulin secretion but also exerts protective effects on β-cells.

#### 5.2.3 Glucose homeostasis

The mechanisms by which BCAAs affect glucose homeostasis are multifaceted. In a cross-sectional study conducted in an Iranian population, dietary BCAA intake correlated significantly negatively with metabolic syndrome, hyperglycemia, and hypertriglyceridemia (85), suggesting that moderate dietary BCAA intake may be protective for glucose homeostasis; elevated circulating BCAAs reflect an underlying state of metabolic dysfunction rather than being causative. Regulation of postprandial glucose fluctuations is also closely related to BCAAs. Protein preloading reduces postprandial glucose fluctuations, partially through delayed gastric emptying and stimulation of GLP-1 secretion (86). This strategy has clinical significance for controlling glucose fluctuations in patients with type 2 diabetes.

The discordance between cross-sectional findings—such as the inverse association between dietary BCAA intake and metabolic syndrome reported in the PERSIAN Kavar cohort (85)—and prospective data showing higher BCAA intake associated with increased T2DM risk (87) illustrates the critical importance of study design, population metabolic status, and background diet in determining the direction of observed associations. In the Iranian cross-sectional study, moderate BCAA intake may reflect overall higher diet quality and adequate protein nutrition in a population without dietary excess; in the Chinese longitudinal data, BCAA increases coincided with a dietary transition toward greater animal protein consumption, confounding BCAA-specific effects with those of saturated fat, heme iron, and other animal product components. These contrasting findings cannot be resolved without studies that rigorously isolate plant-derived from animal-derived BCAA sources and control for background dietary patterns.

#### 5.2.4 Disease progression delay

BCAA metabolism control might be involved in prediabetes treatment. Five BCAAs and aromatic amino acids (isoleucine, leucine, valine, tyrosine, and phenylalanine) were strongly related to future risk for diabetes (39). Three amino acids together could predict the future development of diabetes. Those in the top quartile had over a 5-fold higher risk. Hence, monitoring and modulating BCAA levels could be used to identify at-risk individuals early on.

### 5.3 Dyslipidemia

#### 5.3.1 Lipid profile alterations

The effects of BCAAs on lipid profiles represent an important area of metabolic syndrome research. Studies in Japanese populations found that plasma BCAA levels are positively correlated with triglycerides and negatively correlated with high-density lipoprotein cholesterol (HDL-C) (88). Elevated BCAA levels increase the risk of metabolic dyslipidemia, particularly for the combined phenotype of elevated triglycerides and reduced HDL-C. A case–control study of the Chinese population showed a positive association between dietary BCAA intake and serum total cholesterol and LDL-C, increasing the risk for dyslipidemia (88). However, this association was modulated by total protein consumption and other dietary variables, indicating that the effects of BCAAs on lipid metabolism may be context-dependent.

The epidemiological associations between plasma BCAA levels and dyslipidemia reported in Japanese (88) and Chinese (89) populations differ substantially in direction and magnitude, likely reflecting differences in the dietary sources of BCAAs (fish and soy-predominant Japanese diet versus an increasingly meat-predominant Chinese dietary transition), baseline metabolic status, and the specific lipid phenotype examined (triglyceride and HDL-C in the Japanese data versus total and LDL-C in the Chinese case–control study). These population-specific patterns highlight that the dyslipidemia risk associated with BCAA intake cannot be generalized across dietary contexts and that the lipid-modulating effects of plant-derived versus animal-derived BCAAs may differ qualitatively—not merely quantitatively—depending on the accompanying dietary matrix.

#### 5.3.2 Molecular mechanisms

The molecular mechanisms involved in the effects of BCAAs on lipid metabolism involve several different signaling pathways, with leucine promoting fatty acid synthesis via the activation of sterol regulatory element-binding protein-1 (SREBP-1) through mTOR signaling; SREBP-1 is one of the key transcriptional regulators of insulin-stimulated fatty acid synthesis (46). AMP-activated protein kinase (AMPK) is another important target for BCAA regulation of fatty acid oxidation, as AMPK activation inhibits acetyl-CoA carboxylase activity, promoting fatty acid *β*-oxidation rather than synthesis (47). Moderate BCAA intake may improve lipid metabolism through AMPK activation, while excessive BCAA intake may inhibit AMPK activity. The contribution of BCAAs to cardiovascular disease (CVD) is multifactorial and encompasses several pathways, including lipid metabolism, vascular function, and cardiac energy metabolism (90).

### 5.4 Hypertension

#### 5.4.1 Blood pressure regulation

A close relationship exists between BCAAs and blood pressure regulation. Epidemiological studies have shown that elevated plasma BCAA levels are significantly associated with an increased risk of developing hypertension. In the PREVEND prospective cohort study, plasma BCAA concentrations were positively associated with the risk of incident hypertension. Each standard deviation increase in BCAAs was linked to an 11% increase in hypertension risk (HR 1.11; 95% CI: 1.02–1.20) (91). In a prospective study of a Chinese population, dietary BCAA intake levels were positively correlated with both systolic and diastolic blood pressure. A nonlinear relationship with hypertension risk was observed (92). Direct evidence supports a causal relationship between BCAAs and blood pressure. Activation of BCAA catabolism using BT2 (a BCKDK inhibitor) reduced systolic, diastolic, and mean arterial pressure by approximately 10 mmHg in wild-type mice. Mendelian randomization analysis revealed that elevated plasma BCAA levels predict higher blood pressure in human populations (49). This blood pressure-lowering effect is independent of nitric oxide (NO) signaling and reflects changes in vascular sensitivity to adrenergic constriction.

The observational evidence linking elevated plasma BCAAs to hypertension risk (91–93) is consistently directional but varies substantially in effect magnitude and the extent to which dietary BCAA source (plant versus animal) is differentiated. The PREVEND cohort finding (HR 1.11 per SD increase in plasma BCAAs) (48) is based on plasma BCAA concentrations as a biomarker of combined dietary intake and metabolic BCAA turnover and cannot be directly correlated with dietary BCAA intake from specific food sources. The nonlinear relationship between dietary BCAA intake and hypertension risk reported in a Chinese population (92) suggests the existence of intake thresholds above which risk increases, but the threshold value was identified in a population with a specific dietary background and may not be applicable to other populations. Neither study was designed to isolate the vascular effects of plant-derived from animal-derived BCAAs.

#### 5.4.2 Vascular function

The impact of BCAAs on vascular function is a crucial mechanism by which BP can be regulated, since increased levels of plasma leucine have been reported in obesity and diabetes and are linked to vascular dysfunctions (48). Of all the BCAAs, leucine specifically blocks NO production from L-arginine in ECs through the activation of glutamine:fructose-6-phosphate aminotransferase (GFAT), which is the limiting step in the biosynthesis of glucosamine, thereby inhibiting NO generation. A cross-sectional study in an Iranian population showed that dietary BCAA intake levels are associated with hypertension risk (93). Participants with hypertension had higher total protein and BCAA intake than those with normal blood pressure.

The BCAA metabolite 3-hydroxyisobutyrate can be secreted into circulation and affect endothelial cell function. It promotes trans-endothelial fatty acid transport (35). Accumulation of this metabolite may lead to lipid deposition in the vascular wall and increased arterial stiffness. One might speculate that intermediate intakes of BCAAs may be beneficial as they enhance endothelial function while reducing oxidative stress; it could also be postulated that dietary sources of BCAAs may be more conducive to maintaining vascular function due to a potential additive effect from other antioxidant compounds present in those foods.

### 5.5 Non-alcoholic fatty liver disease

#### 5.5.1 Hepatic lipid accumulation

Non-alcoholic fatty liver disease (NAFLD) is the most common chronic liver disease globally, with a prevalence of approximately 30%. It increased significantly from 25.26% in 1990–2006 to 38.00% in 2016–2019 (94). Defective BCAA metabolism could represent a key element in the pathogenesis of NAFLD. Pathological changes affecting BCAA metabolism occur in conditions such as obesity and T2DM. The main factors involved are pro-inflammatory cytokines, lipotoxicity, and reduced levels of the anti-diabetic hormone adiponectin, which cause excess storage of BCAAs and their downstream metabolites in metabolic tissues and blood circulation (14). This can disrupt insulin signaling, inhibit lipogenesis, and induce inflammatory responses, promoting the development of hepatic steatosis.

#### 5.5.2 Hepatic inflammation and fibrosis

BCAA metabolic regulation may influence the progression of NAFLD to non-alcoholic steatohepatitis (NASH) and hepatic fibrosis. Pharmacological activation of BCAA catabolism improves metabolic parameters in high-fat diet mice, including glucose tolerance and hepatic steatosis (95). Supplemental BCAAs have also shown benefits in ameliorating liver function and prognosis in cirrhotic patients (96). However, in the early stages of NAFLD, high levels of BCAAs can aggravate insulin resistance and hepatic steatosis; thus, BCAA intake should be tailored according to the stage of the disease as well as the metabolic state. Plant-derived BCAAs may have unique advantages in NAFLD prevention and treatment due to their synergistic effects with plant fiber and polyphenolic compounds.

## 6 Comparative analysis of plant-derived and animal-derived branched-chain amino acids

### 6.1 Compositional differences

#### 6.1.1 Nutrient composition comparison

There are major differences between plant and animal sources of protein regarding EAA content and composition. A systematic review of commercially available plant protein isolates using UPLC–MS/MS for determination of amino acid composition has been conducted (18). The examined plant proteins included oat, lupin, wheat, hemp, microalgae, soy, brown rice, pea, corn, and potato. Animal proteins included milk, whey, casein, and egg. Plant proteins such as oat (21%), lupin (21%), and wheat (22%) had significantly lower EAA content compared to animal-derived proteins. Whey protein contained up to 43% EAA, milk protein 39%, casein 34%, and egg protein 32%.

The amount of leucine, another essential amino acid found in various plant proteins, also varied widely, ranging from 5.1% in hemp protein to 13.5% in corn protein. Leucine was present at levels of 9.0% in milk protein, 7.0% in egg protein, and 7.6% in muscle protein. In general, plant proteins had a lower content of methionine (1.0 ± 0.3%) and lysine (3.6 ± 0.6%) compared to animal proteins (methionine 2.5 ± 0.1%, muscle protein methionine 2.0%, lysine 7.8%). It is worth noting that potato protein was the only plant protein source that met all WHO/FAO/UNU EAA requirements. To obtain a similar amount of leucine (2.7 g) or EAAs (10.9 g) as found in 25 g of whey protein, one would need to consume 20–54 g of other plant-based proteins: for example, 31 g of corn protein powder and/or 105 g of hemp protein powder. From these numbers, we can see significant variance among different plant proteins. A combination of multiple plant proteins, along with a plant–animal protein blend approach, could yield an amino acid profile similar to that of animal protein.

#### 6.1.2 Impact of anti-nutritional factors

Plant proteins often contain some antinutritional factors, including phytic acid, tannins, and protease inhibitors, which can decrease the digestion rate and uptake efficiency of proteins. The levels of these antinutrients in foodstuffs can be significantly reduced through processing technologies such as fermentation, heat treatment, and enzymatic hydrolysis, thereby increasing the bioavailability of plant protein.

### 6.2 Metabolic health implications

#### 6.2.1 Epidemiological evidence

Large-scale epidemiological studies have provided important evidence regarding the differential health effects of plant versus animal protein. A systematic review and dose–response meta-analysis included 32 prospective cohort studies involving 715,128 participants (97). Follow-up periods ranged from 3.5 to 32 years. The analysis documented 113,039 deaths (16,429 from cardiovascular disease, 22,303 from cancer).

Total higher protein intakes were associated with a lower risk of death from any cause (pooled HR: 0.94; 95% CI: 0.89–0.99; *I*^2^ = 58.4%). Even more important is that plant protein intake was linked to a lower risk of all-cause mortality (pooled HR 0.92, 95% CI 0.87–0.97; *I*^2^ = 57.5%, indicating moderate heterogeneity) and a reduced risk of cardiovascular disease mortality (pooled HR 0.88, 95% CI 0.80–0.96; *I*^2^ = 63.7%) (97). The high *I*^2^ values and broad confidence intervals across included cohorts indicate substantial between-study heterogeneity, and the modest magnitude of effect (approximately 8–12% risk reduction) should be interpreted in the context of the observational study design and probable residual confounding from dietary pattern complexity (pooled HR 0.88; 95% CI 0.80–0.96, *I*^2^ = 63.7%). However, it was not significantly related to cancer mortality (97). The dose–response analyses suggested that every 3% increase in the proportion of energy from plant protein consumed per day was associated with an approximately 5% lower risk for all-cause mortality.

The NIH-AARP Diet and Health cohort study analyzed 16-year follow-up data from 416,104 participants (98). In this large prospective cohort, substituting plant protein for animal protein (providing 3% of energy) was associated with approximately a 10% lower all-cause mortality risk and an 11–12% lower cardiovascular disease mortality risk (98). However, as with all observational data, residual confounding by overall dietary pattern quality, physical activity, and socioeconomic status cannot be excluded; thus, these findings should be interpreted as hypothesis-generating rather than establishing direct causality.

An umbrella review included 37 meta-analyses regarding diet and the incidence risk of T2D (99). The replacement of animal-based foods with plant-based foods was associated with a significant reduction in CVD risk, type 2 diabetes mellitus, and all-cause mortality. One systematic review assessed the effect of animal versus plant protein intake in relation to the risk of CVD and type 2 diabetes (100). The study included a total of 13 RCTs and 7 cohorts. Replacing animal proteins with plant-based proteins lowered blood cholesterol, deaths from heart disease, and the rate of type 2 diabetes.

#### 6.2.2 Intervention study evidence

Equally important are studies related to the effect of protein sources on maintaining muscular health. Nine studies involving 266 people were included in one meta-analysis (101) comparing the effects of either soy or animal protein (whey, beef, milk, dairy) when combined with resistance exercise for strength and lean body mass gain. Five studies compared whey to soy protein, while four studies compared soy protein with other animal proteins (beef, dairy, or milk).

Both whey protein and soy protein supplementation combined with resistance training resulted in a significant increase in strength. There was no difference between the groups (bench press: *χ*^2^ = 0.02, *p* = 0.90; squat: *χ*^2^ = 0.22, *p* = 0.64) (101). Neither whey nor soy protein alone had a significant impact on changes in lean body mass, with no group difference observed (*χ*^2^ = 0.00, *p* = 0.96). In comparisons of soy protein with other animal proteins, each group showed significant increases in strength and lean body mass, with no significant differences between groups (bench press: *χ*^2^ = 0.02; squat: *χ*^2^ = 0.78, *p* = 0.38; lean body mass: *χ*^2^ = 0.06, *p* = 0.80). This suggests that with sufficient protein intake and resistance exercise, the strength and muscle gains from soy protein are comparable to those from whey or other animal proteins.

### 6.3 The BCAA paradox and its mechanisms

#### 6.3.1 BCAAs and metabolic risk

The branched-chain amino acids (BCAAs) present an interesting paradox. While these amino acids have been shown to positively contribute to muscle protein accretion and the regulation of energy homeostasis, elevated circulating BCAAs are strongly related to higher risks for obesity, insulin resistance, and type 2 diabetes. This paradox was elegantly explained in one study (102). Obese patients exhibited a “BCAA-related metabolic signature,” which consisted of increased plasma levels of BCAAs, along with their metabolites such as C3 or C5 acylcarnitines; this signature correlated with an insulin-resistant state independently of BMI. This implies that BCAA metabolic dysregulation is central to the pathogenesis of metabolic syndrome (102).

In a recent extensive review, we discussed in detail the potential mechanisms involved in the BCAA paradox (12). While BCAAs were frequently found to exert anti-obesity effects in rodents, obese individuals generally display higher circulating concentrations of BCAAs, which are associated with a poor metabolic profile and the future development of insulin resistance or type 2 diabetes (12). Two main mechanistic hypotheses have emerged. First, leucine-induced persistent activation of mTORC1 might result in premature uncoupling of the downstream insulin signaling cascade. Second, the BCAA dysmetabolism model suggests that the accumulation of toxic intermediate metabolites (not the BCAAs per se) can drive *β*-cell mitochondrial dysfunction, stress signaling, and apoptosis related to type 2 diabetes (12). Additionally, insulin resistance can contribute to the development of aminoacidemia through increased protein breakdown, which is inhibited by insulin, and can also interfere with the efficient oxidative metabolism of BCAAs in some tissues.

The results here have implications for the differential health effects of animal versus plant proteins. The former tends to be higher in BCAAs, and prolonged high consumption could lead to a chronic rise in circulating BCAA concentrations. Conversely, the relatively low BCAA content of plant proteins might aid in maintaining BCAA metabolic homeostasis.

#### 6.3.2 Isoleucine restriction and health protection

More recently, however, it has been proposed that isoleucine might be the most important of the three BCAAs when considering metabolic health. In genetically heterogeneous UM-HET3 mice, dietary isoleucine restriction (67% reduction) extended median lifespan by 33% in males and 7% in females, accompanied by improvements in body composition, glycemic control, and frailty indices (103). A subsequent study demonstrated that isoleucine restriction initiated late in life (at 20 months, approximately equivalent to 60 human years) similarly improved metabolic parameters and some aging biomarkers, though with mixed effects on grip strength and cardiac function (104). These are striking preclinical findings that represent an important hypothesis for translational geroscience research. However, several critical caveats must be explicitly stated: (i) rodent lifespan extension does not reliably predict human longevity outcomes, as evidenced by the poor track record of numerous anti-aging interventions that succeeded in mice but failed in humans; (ii) a 67% isoleucine restriction represents a severe dietary constraint far beyond what is achieved by replacing animal protein with plant protein in typical human diets; (iii) no human clinical trial has yet tested the effects of isoleucine restriction on aging, longevity, or metabolic health. The statement that “similar interventions might promote healthy aging among older humans” from the original text has been removed as it constitutes direct extrapolation from animal data to human clinical recommendations, which is not currently justified by available evidence.

#### 6.3.3 Gut microbiota-mediated mechanisms

Differential effects of animal versus plant protein on gut microbiota also constitute an important factor in explaining their differing health effects. L-carnitine is abundant in red meat and can be metabolized by gut microbiota to trimethylamine (TMA), which is subsequently oxidized by hepatic flavin monooxygenases to the pro-atherogenic metabolite trimethylamine N-oxide (TMAO) (105). Omnivores generated much higher levels of TMAO through microbiota-dependent means after consuming L-carnitine than did vegetarians or vegans, while the presence of certain bacterial taxa in human feces correlated with plasma TMAO levels and dietary status.

Among patients undergoing cardiac assessment (*n* = 2,595), plasma L-carnitine levels were predictive of the prevalence risk of cardiovascular diseases and risk of MACE (myocardial infarction, stroke, or death). However, this was only true for those with simultaneously high TMAO concentrations (105). In mice, chronic high-dose dietary carnitine significantly altered the composition of the cecal microbiota, dramatically enhancing the production of TMA and TMAO and accelerating atherogenesis. However, none of these effects were observed if the gut microbiota were also inhibited at the same time. In mice with intact gut microbiota, supplementation with TMAO, carnitine, or choline all reduced *in vivo* reverse cholesterol transport.

**Figure 5:**
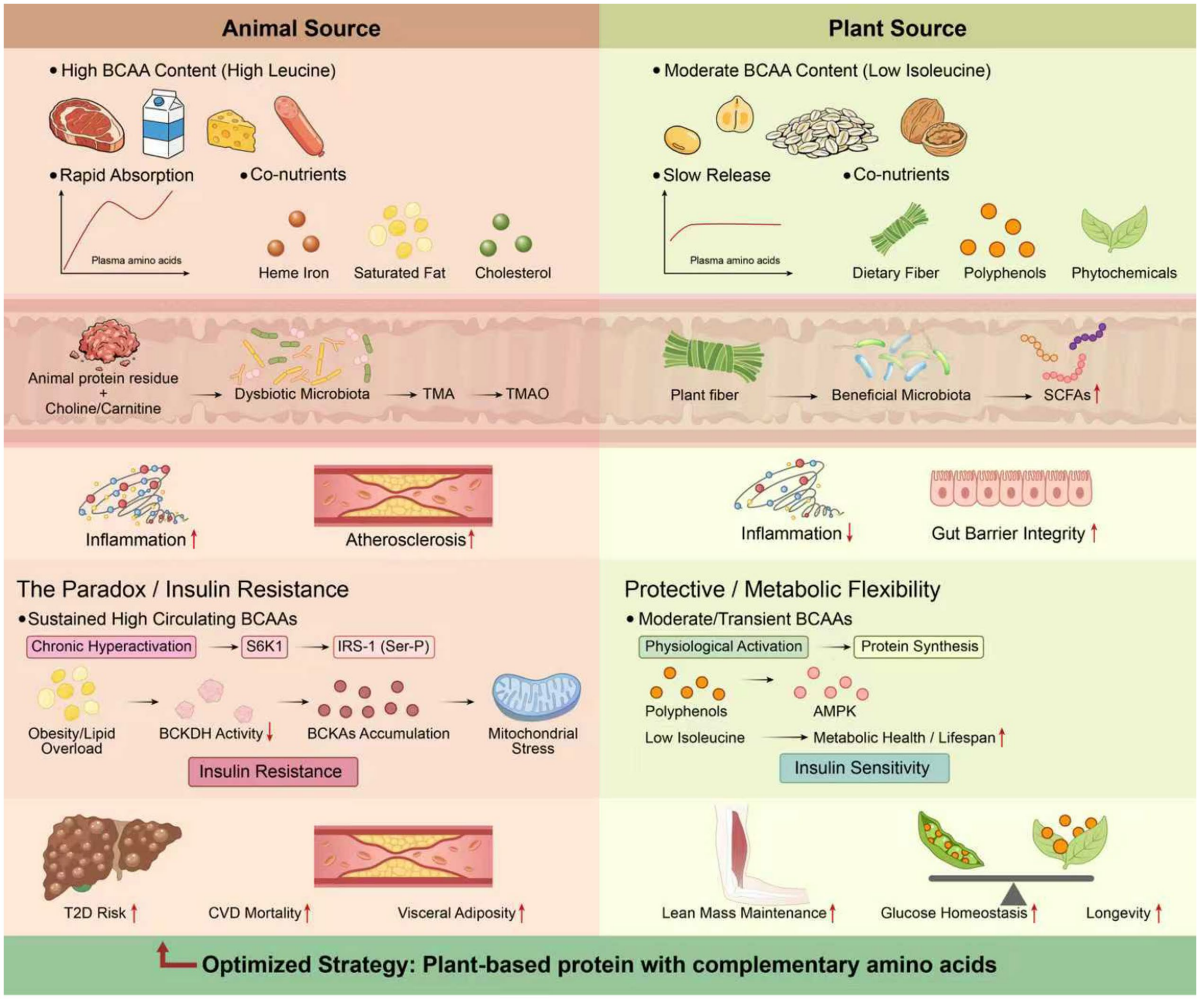
Differing metabolic effects from plant vs. animal-derived BCAAs. Source and matrix determinant model for determining metabolic impact. Rapid absorption and plasma spikes occur with animal-derived BCAAs, accompanied by heme iron and saturated fats, which cause gut dysbiosis, TMAO generation, chronic mTORC1 hyperactivation, IRS-1 dysfunction, and BCKA accumulation—associated with T2D and CVD risk. BCAAs from plant sources show a slow-release kinetics with fiber and polyphenol, favorable microbiota, SCFA production, physiological mTORC1 activation, and AMPK-mediated metabolic flexibility—promoting glucose homeostasis, lean mass, and longevity. Abbreviations: TMAO, trimethylamine N-oxide; IRS-1, insulin receptor substrate-1; BCKAs, branched-chain α-keto acids; T2D, type 2 diabetes; CVD, cardiovascular disease; SCFA, short-chain fatty acid; AMPK, AMP-activated protein kinase. Symbols: → activation/promotion; ⊣ inhibition.

The results indicate that gut microbiota may be involved in the well-documented relationship between high red meat consumption and CVD risk. The integration of the above content is illustrated in Figure 5, further clarifying the matrix effect and the mechanism behind the “BCAA paradox,” ultimately summarizing that the differential metabolic fate of BCAAs from different sources is due to variations in absorption kinetics and accompanying nutrients.

### 6.4 Practical recommendations

#### 6.4.1 Protein requirements for special populations

Protein requirements vary among different populations. A position paper indicated that emerging evidence suggests older adults require more dietary protein than younger individuals (106). This supports good health, promotes recovery from illness, and maintains functionality. Older adults need to compensate for age-related changes in protein metabolism, which include higher splanchnic extraction and diminished anabolic responses to ingested protein. They require more protein to offset the inflammatory and catabolic conditions accompanying chronic and acute diseases associated with aging (106). The expert group recommended that healthy older adults (>65 years) achieve a daily protein intake of 1.0–1.2 g/kg body weight to help maintain and regain lean body mass and function (106). Additionally, aerobic and resistance exercise was recommended for older adults at individualized levels that are safe and tolerable.

Consensus recommendations further stated that: (a) for healthy older adults, diet should provide at least 1.0–1.2 g protein/kg body weight/day; (b) for older adults who are malnourished or at risk of malnutrition due to acute or chronic illness, diet should provide 1.2–1.5 g protein/kg body weight/day. Those with severe illness or injury may require higher intake; (c) all older adults should maintain daily physical activity or exercise (resistance training, aerobic exercise) as long as possible (107).

#### 6.4.2 Protein combination strategies

The individual plant proteins may have a relative deficiency of some EAAs. Using combinations of various plant proteins, as well as mixing plant and animal protein, are techniques to optimize the amino acid profile (18). For instance, legume protein is high in lysine and low in methionine, while cereal protein has the reverse characteristics; combining the two results in amino acid complementation. Mixtures of different plant protein isolates or combinations of plant and animal proteins could also offer a protein profile that closely resembles what we typically find in animal proteins (18).

#### 6.4.3 Individualized nutrition

Protein source selection should consider an individual’s overall metabolic state, desired health outcomes, dietary habits, and sustainability considerations. In populations susceptible to developing IR or MS, appropriate increases in the share of plant protein may help improve metabolic health. For seniors or highly active individuals (e.g., athletes), sufficient total protein consumption is even more important. Provided that there’s enough protein intake with simultaneous resistance training, the type of protein has a relatively insignificant influence on muscular strength and lean body mass accretion (101).

## 7 Clinical evidence and human studies

### 7.1 Observational studies

Before reviewing individual observational studies, it is important to note that the epidemiological evidence base on plant protein, BCAAs, and metabolic syndrome is characterized by substantial heterogeneity in study design, population characteristics, dietary assessment methodology, exposure definition, outcome measurement, and statistical adjustment strategies. A structured summary of key observational studies, including effect sizes, follow-up duration, background diet characteristics, and methodological limitations, is provided in Table 3. Readers are encouraged to consult Table 3 alongside the narrative review to contextualize the effect estimates discussed herein.

**Table 3:** Structured summary of key observational studies on plant protein/BCAA intake and metabolic syndrome components.

Study (Author, Year)	Design	Population (*n*, country)	Exposure definition	Follow-up/study period	Background diet	Primary outcome	Key association (95% CI or *p* value)	Confounders adjusted	Key methodological limitations
Wang et al. (39)	Prospective cohort	*n* = 2,422 normoglycemic individuals, United States	Plasma BCAA metabolomic profiling (isoleucine, leucine, valine, tyrosine, phenylalanine)	12-year follow-up	Framingham Heart Study habitual diet (mixed Western)	Incident T2DM (*n* = 201 cases)	Top vs. bottom quartile of 3-AA signature: >5-fold higher T2DM risk	Age, sex, BMI, fasting glucose, lipids	Plasma BCAAs reflect combined diet + metabolism; reverse causation possible; dietary BCAA intake not directly measured; predominantly white participants
Naghshi et al. (97)	Dose–response meta-analysis of prospective cohorts	32 cohorts, *n* = 715,128, multinational	Dietary plant protein intake (% energy, FFQ or diet history)	3.5–32 years	Mixed (Western, Asian, Mediterranean populations)	All-cause, CVD, cancer mortality	Plant protein: HR 0.92 (95% CI: 0.87–0.97) all-cause; HR 0.88 (95% CI: 0.80–0.96) CVD mortality	Age, sex, BMI, smoking, physical activity, energy intake	*I*^2^ = 57.5% (all-cause), 63.7% (CVD): substantial heterogeneity; FFQ measurement error; residual confounding by overall diet quality; no BCAA-specific assessment
Huang et al. (98)	Prospective cohort (NIH-AARP)	*n* = 416,104, United States	Substitution of plant for animal protein (3% of energy, via FFQ)	16 years	US habitual diet (predominantly Western)	All-cause and CVD mortality	All-cause: −10%; CVD: −11–12% risk reduction with plant protein substitution	Age, sex, race, education, smoking, physical activity, BMI, energy intake, other dietary factors	FFQ-based dietary assessment (measurement error); predominantly older US adults; substitution model assumes constant total protein; no plasma BCAA measurement
Flores-Guerrero et al. (91)	Prospective cohort (PREVEND)	*n* = not fully specified, Netherlands	Plasma BCAA concentration	Not clearly specified	Dutch habitual diet	Incident hypertension	Each SD increase in BCAAs: HR 1.11 (95% CI: 1.02–1.20)	Age, sex, BMI, eGFR, physical activity, dietary factors	Plasma BCAAs as exposure (not dietary intake); single measurement point; reverse causation possible; Dutch population limits generalizability
Glenn et al. (110)	Prospective cohort (NHS, HPFS, NHS-II)	*n* > 200,000, United States	Plant-to-animal protein ratio (FFQ)	Up to 30 years	US habitual diet	CVD risk	Higher plant-to-animal protein ratio is significantly associated with a lower CVD risk (HR not fully specified in text)	Standard CVD risk factors, dietary covariates	FFQ measurement error; US-specific dietary patterns; surrogate exposure (ratio) not equivalent to absolute BCAA intake
Huo et al. (113)	Longitudinal cohort (CHNS)	*n* = not fully specified, China	Healthful plant-based diet index (hPDI)	Multiple survey waves	Chinese habitual diet	Metabolic syndrome prevalence	Higher hPDI independently associated with a lower MetS prevalence; BMI as a partial mediator	Age, sex, urban/rural, physical activity, smoking, energy intake	Self-reported dietary intake; cross-cultural dietary assessment challenges; hPDI is composite index, not BCAA-specific; MetS definition may vary
Nikparast et al. (114) (meta-analysis of observational studies)	Systematic review and dose–response meta-analysis	7 cross-sectional + 1 prospective cohort	hPDI score	Variable	Mixed (predominantly Asian and Western)	Metabolic syndrome risk	Higher hPDI: 19% lower MetS risk (ES = 0.81, 95% CI: 0.67–0.97)	Variable across studies	Predominantly cross-sectional studies (no temporality); heterogeneous MetS definitions; hPDI not BCAA-specific; publication bias possible
Yu et al. (87)	Cross-sectional + prospective cohort (CHNS)	Multiple surveys 1997–2015, China	Dietary BCAA intake (food composition database)	1997–2015 (trend analysis)	Chinese habitual diet (evolving)	T2DM risk	BCAA ≥14.01 g/day associated with higher T2DM risk; highest BCAA intake from red meat, poultry, fish	Age, sex, urbanization, physical activity, energy intake	Cross-sectional design for most analyses; evolving Chinese diet limits temporal comparisons; animal-source BCAAs predominate in high-intake group (confounded by food source)
Shojaei-Zarghani et al. (85)	Cross-sectional (PERSIAN Kavar cohort)	*n* = not fully specified, Iran	Dietary BCAA intake (FFQ)	Cross-sectional	Iranian habitual diet	Metabolic syndrome, hyperglycemia, hypertriglyceridemia	Higher dietary BCAA intake inversely associated with MetS odds	Age, sex, BMI, physical activity, total energy intake	Cross-sectional design (no causality); FFQ measurement error; Iranian population limits generalizability; does not distinguish plant vs. animal BCAA sources
Ruiz-Canela et al. (118)	Nested case–cohort (PREDIMED)	PREDIMED sub-cohort, Spain	Plasma BCAAs (LC–MS) + Mediterranean diet intervention	~5 years	Mediterranean diet (intervention) vs. low-fat control	Incident T2DM	Mediterranean diet + EVOO attenuated BCAA–T2DM association; reduced circulating BCAAs	Standard T2DM risk factors, dietary adherence	Nested observational design within RCT; plasma BCAAs as mediator/biomarker; causal pathway from diet to BCAAs to T2DM not fully established; Spanish population only
Song et al. (147)	Prospective cohort (NHS + HPFS)	*n* = 131,342, United States	Plant vs. animal protein intake (% energy, FFQ)	Long-term follow-up (>20 years)	US habitual diet	All-cause and cause-specific mortality	Plant protein: HR 0.90 (95% CI: 0.86–0.95) per 10% energy increment; substitution of processed red meat with plant protein associated with lower mortality	Multiple lifestyle and dietary covariates	FFQ measurement error; residual confounding; US-specific diet; no BCAA-specific quantification

FFQ, Food frequency questionnaire; CVD, Cardiovascular disease; T2DM, Type 2 diabetes mellitus; MetS, Metabolic syndrome; BCAA, Branched-chain amino acid; hPDI, Healthful plant-based diet index; HR, Hazard ratio; ES, Effect size; CI, Confidence interval; SD, Standard deviation; eGFR, Estimated glomerular filtration rate; EVOO, Extra virgin olive oil; LC–MS, Liquid chromatography–mass spectrometry; CHNS, China Health and Nutrition Survey; PREVEND, Prevention of REnal and Vascular ENd-stage Disease; PREDIMED, Prevención con Dieta Mediterránea; NHS, Nurses’ Health Study; HPFS, Health Professionals Follow-up Study; NIH-AARP, National Institutes of Health–AARP Diet and Health Study. All associations are from observational studies and do not establish causality. Residual confounding by overall dietary pattern quality, socioeconomic status, and lifestyle factors cannot be excluded.

In synthesizing the clinical evidence presented in this section, we have adopted a hierarchical approach to evidence appraisal. Randomized controlled trials (RCTs) and their meta-analyses are treated as the highest level of evidence for causal inference, followed by prospective cohort studies for long-term associations, and cross-sectional studies for hypothesis generation only. Preclinical data (cell-based or rodent studies) discussed elsewhere in this review are not conflated with human clinical evidence herein. Where available, GRADE certainty ratings from included systematic reviews are reported alongside effect estimates. Risk of bias for individual RCTs was assessed using the Cochrane Risk of Bias 2.0 (RoB 2.0) framework (108), and for observational studies using the Newcastle-Ottawa Scale (109). A structured summary of the key studies, including design, sample characteristics, intervention duration, primary outcomes, and effect sizes, is provided in Table 4.

**Table 4:** Summary of key clinical studies on plant-derived proteins/BCAAs and metabolic syndrome components: study design, effect sizes, and risk of bias.

Study (First Author, Year)	Study design	Sample size	Intervention/exposure	Duration	Primary outcome	Key effect size/result	Risk of bias*
Babault et al. (19)	RCT, double-blind, placebo-controlled	*n* = 161 (males, 18–35 y)	Pea protein (25 g × 2/d) vs. whey protein vs. placebo + resistance training	12 weeks	Biceps brachii muscle thickness	Pea vs. placebo: +20.2 ± 12.3% vs. +8.6 ± 7.3% (*p* < 0.05); pea vs. whey: NS	Low–Moderate
McKendry et al. (20)	RCT, parallel-group	*n* = 31 (males, mean 72 ± 4 y)	Whey, pea, or collagen protein (50 g/d additional) vs. RDA control	7-day supplemental phase	Integrated myofibrillar protein synthesis (MPS) rate	Whey: 1.59 ± 0.11%/d; Pea: 1.59 ± 0.14%/d; both vs. control *p* < 0.001; collagen: NS	Low–Moderate
Kahleova et al. (17)	RCT, parallel-group	*n* = 75 overweight adults	Plant-based (PB) diet vs. control diet	16 weeks	Fat mass, insulin resistance	Lower leucine intake correlated with lower fat mass (*r* = +0.40, *p* < 0.001); lower histidine with lower IR (*r* = +0.38, *p* = 0.003)	Moderate
Hanick et al. (124)	RCT	*n* = not specified (Marshallese adults with T2DM)	Whole-food plant-based diet (WFPBD) + exercise vs. standard medical care	24 weeks	HbA1c	Intervention group reduced HbA1c by 1.3% more than controls (14 mmol/mol)	Moderate
Guest et al. (125)	Systematic review and meta-analysis of RCTs	7 RCTs, *n* = 770 patients with T2DM	Vegetarian/vegan dietary pattern	Median ≤24 weeks	HbA1c, BMI	HbA1c: MD = −0.40% (95% CI: −0.59 to −0.21); BMI: MD = −0.96 kg/m^2^ (95% CI: −1.58 to −0.34)	Moderate (GRADE: moderate certainty)
Termannsen et al. (126)	Systematic review and meta-analysis of RCTs	8 RCTs, *n* = 716	Plant-based diet	Variable	HOMA-IR, fasting insulin	HOMA-IR: −0.97 (95% CI: −1.60 to −0.34); fasting insulin reduced	Moderate (GRADE: low–moderate certainty)
Naghshi et al. (97)	Systematic review & dose–response meta-analysis	32 prospective cohorts, *n* = 715,128	Plant protein intake (dietary)	3.5–32 years follow-up	All-cause, CVD, cancer mortality	Plant protein: HR 0.92 (95% CI: 0.87–0.97) for all-cause; HR 0.88 (95% CI: 0.80–0.96) for CVD mortality	Low–Moderate
Huang et al. (98)	Prospective cohort (NIH-AARP)	*n* = 416,104	Substitution of plant for animal protein (3% of energy)	16 years	All-cause and CVD mortality	All-cause: −10%; CVD: −11–12% risk reduction	Low
Depommier et al. (62).	RCT, double-blind, placebo-controlled	*n* = 32 overweight/obese adults	Pasteurized *Akkermansia muciniphila* supplementation	3 months	Insulin sensitivity, cholesterol	Insulin sensitivity: +28.62 ± 7.02% (*p* = 0.002); total cholesterol: −8.68 ± 2.38% (*p* = 0.02)	Low
Ooi et al. (79)	RCT	*n* = 132 overweight/obese	BCAA supplementation vs. normal protein vs. high protein (energy restriction)	16 weeks	Lean mass, body weight	Weight loss ~7.97% across all groups; BCAA group tended to preserve lean mass more; NS difference	Moderate–High
Messina et al. (101)	Meta-analysis of RCTs	9 RCTs, *n* = 266	Soy protein vs. animal protein + resistance exercise	Variable	Muscle strength, lean body mass	No significant difference between soy and whey/animal protein (bench press: *p* = 0.90; lean mass: *p* = 0.96)	Moderate
Ruiz-Canela et al. (118)	Nested case–cohort (PREDIMED)	Sub-cohort of PREDIMED trial	Mediterranean diet + extra virgin olive oil	~5 years (PREDIMED)	Circulating BCAAs, T2D risk	Mediterranean diet attenuated BCAA–T2D association; reduced circulating BCAA levels	Low–Moderate
Wang et al. (39)	Prospective cohort	*n* = 2,422 normoglycemic individuals; 201 developed T2D	Plasma BCAA metabolomic profiling	12-year follow-up	Incident T2D	Top quartile of 3-AA signature: >5-fold higher T2D risk	Low

RCT, Randomized controlled trial; T2DM, Type 2 diabetes mellitus; CVD, Cardiovascular disease; HbA1c, Glycated hemoglobin; HOMA-IR, Homeostatic model assessment of insulin resistance; BMI, Body mass index; MPS, Muscle protein synthesis; NS, Not significant; HR, Hazard ratio; MD, Mean difference; CI, Confidence interval. Risk of bias: Low, Adequate randomization, allocation concealment, blinding, and complete outcome data; Moderate, One or more domains with unclear or some concern; High, Serious risk of bias in one or more domains. For observational studies, risk of bias assessed by Newcastle-Ottawa Scale (stars ≥7 = low risk).

#### 7.1.1 Cohort studies

Important epidemiologic evidence has been provided by large-scale prospective cohort studies showing how the effects of dietary branched-chain amino acids from plants differ from those derived from animals in human metabolism. The NHS followed more than 200,000 participants over a period of up to 30 years (110). The proportion of plant protein and animal protein intake gradually changed from around 1:3 to 1:2. A greater plant-to-animal protein ratio was significantly associated with lower CVD risk.

The same result is confirmed by the European Prospective Investigation into Cancer and Nutrition (EPIC). The EPIC-Italy study included 45,009 adults from Italy, with a median follow-up time of 15.2 years (111). In the EPIC-Italy cohort (*n* = 45,009; median follow-up 15.2 years), the replacement of animal proteins with plant proteins was associated with a 47% lower risk of cardiovascular death (HR = 0.47; 95% CI: 0.24–0.92) (3). This association should be interpreted with caution given the observational design, the potential for dietary misclassification through food frequency questionnaires, and the moderate risk of bias inherent in cohort studies of this nature (Newcastle–Ottawa Scale assessment: 6–7 stars). The protective effect for CRC incidence was evident only among those with ≥ 1 unhealthful lifestyle risk factor coupled with low adherence to the Mediterranean Diet. In the EPIC-Heidelberg cohort study, consumption of red or processed meat was positively associated with all-cause mortality, cardiovascular mortality, and cancer-related mortality (HR range 1.25–1.76) (112). Consumption of poultry, milk, or cheese was protective against mortality. However, after full adjustment for lifestyle factors, most associations became nonsignificant, indicating the important role of confounders in protein–health associations.

The CHNS provided an opportunity to study Asians, as this prospective cohort study of Chinese adults reported that a greater healthful plant-based diet index (hPDI) was independently linked with lower prevalence of MS (113), particularly concerning visceral fat accumulation. BMI could mediate the effect of hPDI on MS. A meta-analysis of seven cross-sectional studies and one prospective cohort study demonstrated trends in dietary BCAA intakes for the Chinese population between 1997 and 2015 (87). Intake of BCAA ≥14.01 g per day was related to higher T2D risk, while high BCAA intake was most closely associated with consumption of red meat, poultry, fish, and seafood.

Regarding the role that plant proteins play in MS risk, data from NHANES 2015–2016 showed that each additional daily serving of healthful plant foods was associated with a 4% lower risk for MS. Additionally, each additional daily serving was associated with a 4% lower risk of elevated waist circumference (WC). This finding was replicated through a dose–response meta-analysis (114). The association between hPDI and the risk of MS indicated that a higher adherence level to the hPDI was related to a 19% decrease in the risk of developing MS (ES = 0.81, 95% CI: 0.67–0.97). Conversely, high adherence to an unhealthy plant-based diet (uPDI) index increased the risk by 27%.

#### 7.1.2 Cross-sectional studies

Other studies assessing the relationship between diet, macronutrients, or amino acid consumption and metabolic biomarkers suggested some positive effects of a diet rich in plant-derived BCAAs on human metabolism. In a case–control study among Chinese individuals suffering from T2DM, complex relationships were observed between BCAA food intake and cardiovascular disease risk (115). High levels of circulating BCAAs correlated directly with increased activity of the mTOR pathway and insulin resistance (IR). Controlled feeding trials showed that sulfur amino acid (SAA) restricted diets can lead to significant reductions in body weight (BW) and waist circumference (WC) after only 4 weeks of intervention (116), as well as improvements in cardiometabolic disease risk biomarkers. Plasma BCAA concentrations were lower with decreasing SAA intakes.

The Mediterranean diet and DASH diet are typical healthy dietary patterns. Their BCAA characteristics have attracted attention. The Atherosclerosis Risk in Communities (ARIC) study identified 27 metabolomic markers associated with the modified MIND diet (117). These provide opportunities for objective assessment of dietary adherence. A nested case–cohort study within the PREDIMED trial found that a Mediterranean diet supplemented with extra virgin olive oil could reduce circulating BCAA levels (118). It also diminished the positive association between BCAAs and type 2 diabetes risk. This suggests that dietary intervention can modulate BCAA-mediated metabolic risk.

It should be emphasized that all cross-sectional studies cited in this section are inherently limited by their inability to establish temporality or causality. The associations between dietary BCAA intake and metabolic biomarkers reported herein may reflect reverse causation (whereby individuals with metabolic dysfunction alter their dietary patterns) or unmeasured confounding. These findings should be considered exploratory and hypothesis-generating, requiring confirmation from prospective or interventional designs.

### 7.2 Randomized controlled trials

The randomized controlled trials reviewed in this section differ substantially in intervention type (free-form BCAA supplementation, protein isolate supplementation, or whole-food dietary pattern intervention), comparator conditions, study duration, population metabolic status, background diet control, and primary outcome selection. A structured summary of key RCTs, including intervention details, effect sizes, and methodological limitations, is provided in Table 5. These methodological differences substantially limit cross-study comparisons and should be considered when interpreting the evidence synthesized in the meta-analyses discussed in Section 7.3.

**Table 5:** Structured summary of key randomized controlled trials on plant-derived proteins/BCAAs and metabolic syndrome components.

Study (Author, Year)	Design	Population (*n*, characteristics)	Intervention	Comparator	Background diet	Duration	Primary outcome	Key effect size (95% CI)	Methodological limitations
Babault et al. (19)	Double-blind RCT, parallel	*n* = 161, healthy males, 18–35 y	Pea protein 25 g × 2/d + resistance training	Whey protein 25 g × 2/d; placebo + resistance training	Ad libitum, uncontrolled	12 weeks	Biceps brachii muscle thickness	Pea vs. placebo: +20.2 ± 12.3% vs. +8.6 ± 7.3% (*p* < 0.05); pea vs. whey: NS (*p* = 0.09)	Only young healthy males; uncontrolled ad libitum diet; muscle thickness by ultrasound (operator-dependent); short duration; no metabolic outcomes
McKendry et al. (20)	RCT, parallel-group	*n* = 31, healthy older males, 72 ± 4 y	Pea, whey, or collagen protein 50 g/d (2 × 25 g at breakfast and lunch)	RDA-level protein control phase (0.8 g/kg/d)	Fixed protein (RDA phase), then supplemental phase	7-day supplemental phase	Integrated myofibrillar protein synthesis (MPS) rate (%/d)	Pea: 1.59 ± 0.14%/d vs. control 1.46 ± 0.10%/d (*p* < 0.001); collagen: NS	Very short duration (7 days); exclusively older males; crossover confounding; stable isotope MPS not measured under free-living conditions; no clinical endpoints
Kahleova et al. (17)	RCT, parallel-group	*n* = 75, overweight adults (BMI 28–40)	Plant-based (PB) diet (ad libitum)	Control diet (no dietary restrictions)	Plant-based vs. unrestricted omnivorous	16 weeks	Fat mass, insulin resistance (HOMA-IR)	Lower leucine intake correlated with lower fat mass (*r* = +0.40, *p* < 0.001); lower histidine with lower IR (*r* = +0.38, *p* = 0.003)	Correlational analysis of amino acid intake, not a BCAA supplementation trial; self-reported dietary intake; open-label (no blinding possible); BCAA-specific attribution confounded by overall dietary pattern change
Hanick et al. (124)	RCT, parallel-group	*n* = not fully reported, Marshallese adults with T2DM	Whole-food plant-based diet (WFPBD) + exercise program	Standard medical care	WFPBD vs. habitual diet	24 weeks	HbA1c	Between-group difference: −1.3% (−14 mmol/mol)	Single ethnic population (Marshallese); co-intervention with exercise prevents isolation of dietary effect; no blinding; medication changes confound glycemic outcomes; BCAA intake not measured separately
Ooi et al. (79)	RCT, triple-arm, parallel	*n* = 132, overweight/obese adults	BCAA supplementation vs. high-protein diet vs. normal-protein diet (all with energy restriction)	Normal protein + energy restriction	Energy-restricted diets, background diet not fully characterized	16 weeks	Lean mass, body weight	Weight loss ~7.97% across all groups; lean mass preservation trend in BCAA group (NS)	High dropout (not reported clearly); BCAA dose not individualized by body weight; background diet not standardized; short-term only; no long-term follow-up
Novin et al. (80)	RCT, parallel-group	*n* = not specified, overweight and obese women	BCAAs + vitamin B6 supplementation	Control (not specified)	Not characterized	Not clearly reported	Leg lean mass, waist-to-hip ratio	Leg lean mass preserved; waist-to-hip ratio improved	Female-only; vitamin B6 co-supplementation prevents BCAA-specific attribution; background diet uncontrolled; limited methodological details reported
Singh et al. (121)	RCT, randomized, comparator-controlled, parallel	*n* = 84 days (*n* not clearly reported), sedentary adults	Pea protein supplementation + resistance training	Whey protein + resistance training; resistance training only	Ad libitum, uncontrolled	84 days (12 weeks)	Muscular power, grip strength, upper and lower limb strength	No significant difference between pea and whey protein groups across all strength measures	Sedentary adult population limits generalizability to athletic populations; ad libitum diet; blinding not clearly described; metabolic outcomes not measured
Loureiro et al. (122)	RCT, double-blind, crossover	*n* = not specified, male soccer athletes	Pea protein supplementation	Whey protein supplementation	Controlled athlete diet	Not clearly reported	Metabolic profile (lipids, glucose, insulin)	No significant difference between pea and whey protein on any metabolic parameter	Crossover design with carryover risk; athlete population limits generalizability to MetS patients; washout period not clearly specified; small sample size
Depommier et al. (62)	RCT, double-blind, placebo-controlled	*n* = 32, overweight/obese adults (no T2DM)	Pasteurized *Akkermansia muciniphila* 10^10^ CFU/d	Placebo	Ad libitum, Mediterranean-style diet	3 months	Insulin sensitivity, total cholesterol, inflammatory markers	Insulin sensitivity: +28.62 ± 7.02% (*p* = 0.002); total cholesterol: −8.68 ± 2.38% (*p* = 0.02)	Small sample (n = 32); proof-of-concept study only; not a dietary BCAA intervention; indirect relevance to plant-derived BCAAs via gut microbiota pathway only
Guest et al. (125) (meta-analysis)	Systematic review and meta-analysis of RCTs	7 RCTs, *n* = 770 patients with T2DM	Vegetarian or vegan dietary pattern	Various comparator diets	Vegetarian/vegan vs. omnivorous	Median ≤24 weeks	HbA1c, BMI	HbA1c: MD = −0.40% (95% CI: −0.59 to −0.21); BMI: MD = −0.96 kg/m^2^ (95% CI: −1.58 to −0.34)	High study heterogeneity (*I*^2^ > 50% for BMI); short median duration; varied definitions of vegetarian/vegan diet; medication confounding not fully addressed; GRADE: moderate certainty
Termannsen et al. (126) (meta-analysis)	Systematic review and meta-analysis of RCTs	8 RCTs, *n* = 716	Plant-based diet	Various comparator diets	Plant-based vs. omnivorous	Variable	HOMA-IR, fasting insulin	HOMA-IR: −0.97 (95% CI: −1.60 to −0.34)	Substantial heterogeneity in dietary protocols; varied duration; BCAA intake not measured; GRADE: low–moderate certainty
Zhou et al. (119) (meta-analysis)	Systematic review and meta-analysis of RCTs	63 RCTs, individuals with metabolic diseases	High-quality protein supplementation (including soy, pea)	Various control conditions	Variable, not standardized	Variable (weeks to months)	SBP, total cholesterol, LDL-C	SBP: −1.42 mmHg; TC: −0.18 mmol/L; LDL-C: −0.16 mmol/L	High heterogeneity across protein types and populations; modest absolute effect sizes; several small trials with unclear allocation concealment; *I*^2^ > 50% for multiple outcomes

RCT, Randomized controlled trial; T2DM, Type 2 diabetes mellitus; MPS, Muscle protein synthesis; HbA1c, Glycated hemoglobin; HOMA-IR, Homeostatic model assessment of insulin resistance; BMI, Body mass index; SBP, Systolic blood pressure; TC, Total cholesterol; LDL-C, Low-density lipoprotein cholesterol; NS, Not significant; MD, Mean difference; CI, Confidence interval; BCAA, Branched-chain amino acid; WFPBD, Whole-food plant-based diet; RDA, Recommended dietary allowance. Effect sizes are reported as published in original studies. Where I^2^ values are reported in meta-analyses, values >50% indicate substantial heterogeneity, and pooled estimates should be interpreted with caution.

#### 7.2.1 Supplementation studies

The studies evaluating the effect of soy protein supplementation provided a simplified view regarding the impact of dietary plant-based protein intake on human metabolism.

A recent systematic review and meta-analysis of 63 RCTs (119) reported that soy protein supplementation lowered systolic blood pressure by −1.42 mmHg, total cholesterol by −0.18 mmol/L, and LDL cholesterol by −0.16 mmol/L. The authors noted that the quality of included RCTs was heterogeneous; several trials had small sample sizes (*n* < 30), short durations (≤8 weeks), and unclear allocation concealment, corresponding to a moderate overall risk of bias. Effect sizes, while statistically significant, are modest in absolute magnitude, and their clinical relevance requires contextual interpretation, particularly in primary prevention settings. The systematic review on the use of soy protein by athletes or other physically active individuals included a total of 19 RCTs (120). It evaluated the effect of soy protein in relation to muscle adaptation, metabolic status, antioxidant capacity, hormonal response, and exercise performance. Soy protein may be used instead of whey protein.

Other studies using peas as a supplementary source of protein have produced equally positive outcomes. A 3-month randomized triple-blind controlled clinical trial comparing pea versus whey supplemented groups was conducted on a sedentary adult population and found that both supplements yielded similar gains in terms of total muscular power (121), grip strength, and lower and upper limb strength in combination with resistance training. The product perception was favorable. A randomized, double-blind, crossover trial with soccer athletes compared the effects of pea versus whey protein on metabolic profile (122). There was no difference between the two.

The potential for an amino acid complementary strategy using mixed plant proteins was also investigated in a study. A mixed dairy and plant protein blend (35% whey, 25% casein, 20% soy, 20% pea) exhibited a more balanced amino acid pattern with a higher chemical score (CS) (123). In terms of dose, study length, and population studied, the results from a meta-analysis demonstrated that the effect of protein supplements on cardiovascular risk factors varies depending on an individual’s health status (119). The antihypertensive effect and lipid-lowering action were more evident among the metabolic disease group.

#### 7.2.2 Whole food interventions

The interventional studies on plant-based diets have provided some indication regarding their clinical application. There was one large randomized controlled trial of a whole-food, plant-based diet in Marshallese adults with T2DM over 24 weeks, where patients were randomized between WFPBD plus exercise and control medical care (124). Compared to controls, the intervention group achieved a between-group difference of 1.3% (14 mmol/mol) in HbA1c reduction (124). This effect size is clinically meaningful (exceeding the 0.5% threshold often considered clinically relevant for glycemic management); however, the generalizability of this finding is limited by the specific ethnic population studied (Marshallese adults), the co-intervention of exercise, and the relatively short 24-week follow-up. The risk of bias was assessed as moderate, primarily due to the inability to blind participants to dietary assignment. The intervention group may be able to reduce medications significantly more compared to controls.

Systematic reviews on vegetarian or vegan studies have provided high-quality evidence. A systematic review and meta-analysis of RCTs from 1998 to May 2023 (including 7 trials with a total of 770 patients with T2DM) (125) found that a vegetarian diet pattern may reduce HbA1c (MD = −0.40, 95% CI: −0.59 to −0.21, moderate-certainty evidence). Body mass index is likely reduced as well (MD = −0.96 kg/m^2^, 95% CI: −1.58 to −0.34). The first PRISMA-compliant systematic review (SR) on plant-based diets and insulin sensitivity markers consisted of 8 RCTs (716 participants) (126). Compared to the controls, plant-based diets lowered HOMA-IR (−0.97, 95% CI: −1.60 to −0.34) and fasting insulin.

### 7.3 Systematic reviews and meta-analyses

Evidence evaluation. Existing evidence must be evaluated through a systematic review: For the Nordic Nutrition Recommendations 2022, 15,090 articles were screened (100). Ultimately, there were 13 RCTs and 7 cohort studies. Eight RCTs had some or high risk of bias, and all seven cohort studies had medium risk of bias. Meta-analysis showed that replacing animal protein with plant protein can protect against total cholesterol (MD = −0.11 mmol/L) and low-density lipoprotein cholesterol (MD = −0.14 mmol/L). This replacement was associated with reduced CVD mortality and T2D incidence. The quality of evidence is limited/suggestive.

Another systematic review and meta-analysis, which included 37 studies based on 24 cohorts (127), also applied the GRADE approach for assessing the certainty of evidence. Replacing processed meat (PM) with nuts or legumes decreased CVD risk. Replacing red meat (RM) with whole grains or nuts decreased T2D risk. Substituting RM or PM with nuts or legumes decreased overall mortality. Heterogeneity observed in this analysis was largely attributable to differences in populations studied, dietary assessment methodologies, and follow-up times. Regarding publication bias, most of the included studies used funnel plots and Egger’s test to evaluate it (128). We did not find any apparent indication of publication bias; however, small sample research can produce unstable effect sizes.

Formal appraisal of evidence certainty using the GRADE framework reveals important gradations across the reviewed outcomes. The evidence for improved glycemic control (HbA1c reduction) with plant-based dietary patterns in T2DM is rated as moderate certainty (125), based on consistent directional effects across multiple RCTs but limited by short trial durations and high participant dropout in some studies. Evidence for reduced HOMA-IR and fasting insulin is rated as low-to-moderate certainty (126), given heterogeneity in dietary protocols and timing of outcome measurements. Evidence for reduced all-cause and CVD mortality with higher plant protein intake derives exclusively from observational cohorts, and no RCT has yet demonstrated a reduction in hard cardiovascular endpoints attributable to plant protein substitution; therefore, this body of evidence is rated as low certainty for causal inference despite consistent epidemiological associations. These GRADE assessments align with those independently reported by Lamberg-Allardt et al. (100) and Szczerba et al. (129), whose umbrella reviews reached similar conclusions.

A quantitative assessment of between-study heterogeneity across the meta-analyses cited in this section reveals a consistent pattern of moderate-to-substantial heterogeneity that warrants explicit acknowledgment. Naghshi et al. (97), the I^2^ for all-cause mortality was 58.4%, and for CVD mortality, it was 63.7%, indicating that more than half of the observed variance in effect estimates is attributable to between-study differences rather than sampling error. Guest et al. (125), heterogeneity in BMI outcomes exceeded *I*^2^ = 50%. Zhou et al. (119), multiple cardiovascular risk factor outcomes exhibited *I*^2^ > 50%. While random-effects models were appropriately used in these meta-analyses to account for heterogeneity, it is important to recognize that random-effects pooling does not eliminate the interpretive challenges posed by high heterogeneity—it simply widens confidence intervals to reflect uncertainty. The clinical implication is that the “average” treatment effect estimated by these meta-analyses may not accurately represent the effect in any specific patient population, and applying pooled estimates to clinical recommendations for metabolic syndrome patients requires cautious, context-sensitive interpretation. Subgroup analyses by geographic region, metabolic status, background diet, and intervention type have been inconsistently reported, and where conducted, frequently lacked statistical power due to small subgroup sample sizes.

### 7.4 Limitations of evidence and research gaps

Despite existing evidence supporting the health benefits of plant-derived protein/BCAAs, several important limitations remain.

Firstly, there is a lack of long-term trials. The vast majority of RCTs do not extend beyond 24 weeks in length (125, 126). This leaves us unable to determine how effective plant-based dietary patterns will be over the longer term concerning chronic disease endpoints. Only one umbrella review was able to include any RCTs that lasted for ≥12 weeks (129). Many more were excluded due to being too short-term, but even then, long-term data on the association of plant-based diets with hard outcomes like diabetes complications and CV events is lacking.

Secondly, there exists a gap between mechanistic and clinical studies. Basic research has demonstrated how BCAAs impact insulin sensitivity and glucose-lipid metabolism through certain pathways (130), including the mTOR signaling pathway and the regulation of BCKDH enzyme activities. Nevertheless, how these effects translate to quantifiable health endpoints during a clinical intervention remains unclear. The connection between circulating levels of BCAAs, a marker of metabolic status, and the intake of these amino acids through diet needs to be clarified through more sophisticated experimental approaches in the future (130).

Thirdly, individual differences are often neglected. Regarding genetic background, BCKDH gene polymorphisms can significantly affect BCAA catabolism efficiency (131). Concerning gut microbiota, different microbial compositions can modulate the intestinal metabolism of BCAAs and their availability to the host (132). In terms of metabolic phenotype, insulin-sensitive individuals and insulin-resistant individuals may exhibit completely different metabolic responses to equivalent BCAA intake (12). Future research needs to integrate multi-omics data to develop precision nutrition strategies.

Last but not least is the issue of dose and proportionality. Analysis of CHNS data found a nonlinear relationship between plant protein consumption and metabolism (133). This implies potential thresholds. There are still some issues that need to be addressed in future research (134), such as the optimal ratio of plant-to-animal protein, the relative contributions from various plant protein sources, and interactions with other dietary components (e.g., dietary fibers, polyphenols).

A fundamental methodological limitation of the current evidence base is the absence of systematic, formal risk of bias assessments across the studies informing this review. The majority of included RCTs were not pre-registered in clinical trial registries, lacked independent data monitoring committees, and employed subjective dietary assessment instruments (food frequency questionnaires, 24-h dietary recalls) as both the exposure measurement tool and the primary intervention fidelity check. This introduces a double layer of measurement error. Furthermore, pooling heterogeneous populations (varying age, BMI, baseline BCAA status, gut microbiota composition, and genetic background) across meta-analyses yields pooled effect estimates that may obscure clinically important subgroup variations. In several key meta-analyses cited in this review (97, 125, 126), I^2^ statistics exceeded 50%, indicating substantial heterogeneity that was not fully explained by pre-specified subgroup analyses. Readers should interpret the pooled estimates with appropriate caution.

### 7.5 Sources of heterogeneity across studies and their implications for evidence synthesis

A critical challenge in synthesizing the clinical and epidemiological evidence reviewed herein is the substantial and often poorly characterized heterogeneity between studies. This heterogeneity operates across at least five distinct dimensions, each of which independently limits the interpretability of pooled estimates and between-study comparisons.

#### 7.5.1 Background diet heterogeneity

The metabolic effects of plant-derived BCAAs are closely intertwined with the broader dietary context, and background diet represents perhaps the most important uncontrolled source of variation between studies. Studies conducted in populations habitually consuming Western dietary patterns (high in saturated fat, refined carbohydrates, and animal protein; low in dietary fiber) cannot be directly compared with studies in populations consuming Mediterranean, Asian, or plant-predominant dietary patterns. For example, the inverse association between dietary BCAA intake and metabolic syndrome reported in an Iranian cohort (85) exists within a dietary context of moderate plant food consumption and relatively low total protein intake, while the positive association between high BCAA intake and T2DM risk identified in Chinese longitudinal data (87) reflects a dietary transition period in which increases in BCAA intake were driven primarily by rising red meat and poultry consumption rather than plant protein. These contextually opposite findings emphasize that the metabolic effect of a given level of BCAA intake cannot be generalized across dietary backgrounds. Furthermore, meta-analyses pooling studies from Western, Asian, Mediterranean, and developing-country populations inevitably generate pooled estimates that obscure clinically important heterogeneity, as evidenced by I^2^ values exceeding 57% in the largest meta-analysis of plant protein and mortality (97). The background diet also determines the co-exposure to fiber, polyphenols, and saturated fat that fundamentally alters how BCAAs are metabolized and their downstream effects on inflammation and insulin signaling, as discussed in Section 6.3.

#### 7.5.2 Study duration heterogeneity

Intervention duration varies significantly across the RCTs included in this review, ranging from 7 days (20) to 24 weeks (124), with the majority falling within the 8–16 week range. This variability is particularly problematic because the metabolic outcomes of interest in MetS—changes in insulin sensitivity, lipid profiles, body composition, and gut microbiota composition—occur on different time scales. Gut microbiota compositional shifts may occur within days to weeks (61), whereas meaningful changes in HbA1c require at least 8–12 weeks of sustained dietary modification, and changes in muscle mass assessed by gold-standard methods require 12–24 weeks of combined dietary and exercise intervention. The observation that HbA1c was significantly reduced in vegetarian/vegan diet RCTs (MD = −0.40%) (125) while body weight and lean mass outcomes showed more modest and heterogeneous effects likely reflects, in part, the differing time constants of these physiological responses rather than true differential efficacy. Long-term outcomes (cardiovascular events, incident diabetes, all-cause mortality) are accessible only through observational cohort studies with follow-up periods of 10–30 years (97, 98), and the inability to reconcile short-term RCT mechanistic data with long-term observational associations represents a fundamental epistemic gap in this field.

#### 7.5.3 BCAA dose and dietary protein form heterogeneity

Studies differ substantially in whether they investigate: (i) supplemental free-form BCAAs (e.g., BCAA powder supplements (80, 82)); (ii) protein-form plant BCAAs delivered through protein isolates (e.g., pea protein isolate (19, 121)); or (iii) whole-food plant-based dietary patterns in which BCAAs are one component among many (17, 124). These three forms are not metabolically equivalent. Free-form BCAAs bypass the digestive rate-limiting steps that characterize intact protein digestion, producing more rapid postprandial plasma BCAA spikes that may differentially activate mTORC1 and insulin signaling compared to the same amino acids delivered within an intact protein matrix (41). Protein isolates, while closer to whole-food conditions than free-form supplements, still lack the dietary fiber and polyphenol co-matrix of whole plant foods that modulate absorption kinetics and gut microbiota interactions. Dosage also varies substantially: supplemental BCAA studies have used doses ranging from approximately 4 g/day to more than 20 g/day, while whole dietary pattern interventions do not specify BCAA dosage at all. This heterogeneity in form and dosage makes comparison across studies fundamentally problematic, and the absence of standardized dietary BCAA quantification using validated metabolomic methods further compounds this limitation.

#### 7.5.4 Metabolic status heterogeneity of study populations

A recurring source of heterogeneity is the wide variation in baseline metabolic status of enrolled participants, ranging from healthy young athletes (122) to overweight but metabolically healthy adults (17), to individuals with established T2DM (124, 125), and to older adults at risk of sarcopenia (20). The metabolic response to equivalent BCAA intake differs substantially across these groups. In metabolically healthy individuals with intact insulin signaling, leucine-stimulated mTORC1 activation promotes anabolism without impairing insulin sensitivity, whereas in individuals with established insulin resistance, the same stimulus may exacerbate S6K1-mediated IRS-1 serine phosphorylation (12). This context-dependency means that a treatment effect (or lack thereof) observed in one population cannot be generalized to another. The meta-analyses available to date (119, 125, 126) have not adequately addressed this through pre-specified metabolic status subgroup analyses, limiting their clinical applicability. Moreover, baseline BCAA catabolic capacity—which varies with BCKDH genetic polymorphisms (131), gut microbiota composition (132), and adipose tissue metabolic status (33)—is rarely characterized, precluding the identification of metabolic responders and non-responders.

#### 7.5.5 Outcome measurement and definition heterogeneity

Substantial heterogeneity exists in how primary outcomes are defined and measured across studies. Insulin resistance is assessed using various methods, including HOMA-IR, hyperinsulinemic–euglycemic clamp (the gold standard), fasting insulin, or HbA1c—methods that differ significantly in sensitivity and physiological meaning. Dietary BCAA intake is quantified through food frequency questionnaires, 24-h dietary recalls, or weighed food records, each presenting distinct measurement error profiles; plasma BCAAs are measured using HPLC, targeted LC–MS/MS, or enzymatic assays, introducing further analytical variability (129). MetS itself is defined using multiple criteria sets (IDF, NCEP-ATP III, AHA/NHLBI harmonized), making it challenging to compare studies that utilize different definitions. Body composition outcomes (lean mass, fat mass) are assessed by DEXA, BIA, or anthropometric proxies, each with varying accuracy. This outcome measurement heterogeneity means that even studies nominally measuring “the same” endpoint often do not measure equivalent constructs, which inflates between-study variance and reduces the interpretability of pooled meta-analytic estimates.

### 7.6 Implications for evidence synthesis

Taken together, these five sources of heterogeneity imply that the pooled effect estimates from existing meta-analyses (97, 119, 125, 126) should be viewed as broad directional signals rather than precise quantitative estimates applicable to specific clinical populations. Future research should prioritize: (i) pre-registration of studies with explicit *a priori* subgroup analyses based on metabolic status, background diet, and BCAA dose; (ii) standardized dietary BCAA quantification using LC–MS/MS-based metabolomics alongside dietary assessment; (iii) longer intervention durations (minimum 12 months for metabolic outcomes, with follow-up on hard clinical endpoints); and (iv) geographically and ethnically diverse study populations to enable assessment of whether dietary background modifies the relationship between plant-derived BCAA intake and metabolic health.

## 8 Safety, bioavailability enhancement, and practical applications

### 8.1 Safety assessment

#### 8.1.1 Safe dosage range

The safety profiles of dietary versus supplemented branched-chain amino acids (BCAAs) differ. Dietary branched-chain amino acids are bound to proteins, resulting in more balanced, physiological metabolism (130). Supplemented free-form BCAAs may cause a sharp rise in circulating BCAA levels, potentially interfering with the transport of aromatic amino acids across the blood–brain barrier.

Regarding Upper Intake Levels (UL), the IOM stated that due to limited dose–response information, no UL for BCAAs was established. In 2019, the German Federal Institute for Risk Assessment (BfR) announced that high dosages of free-form BCAAs through dietary supplements can be harmful to health, and total intake from preparations must not exceed 8.2 g/day. This calculation is based on a body mass of 60 kg.

Special populations require particular attention. Patients with maple syrup urine disease have genetic defects in the branched-chain *α*-keto acid dehydrogenase complex. BCAAs and their metabolites accumulate, leading to severe neurotoxicity. These patients require lifelong strict dietary BCAA restriction (135).

#### 8.1.2 Long-term safety

Regarding renal function, a one-year randomized crossover trial demonstrated specific findings (136). Healthy resistance-trained males consumed a high-protein diet (2.51–3.32 g/kg/d) and showed no harmful effects on blood lipids, hepatic function, or renal function parameters. A systematic review and meta-analysis further confirmed these findings (137). In healthy adults, higher protein intake (≥1.5 g/kg body weight or ≥20% of energy intake) was compared with normal or lower protein intake. No significant effect was observed on changes in glomerular filtration rate (GFR).

Concerning bone health, an expert consensus paper (endorsed by ESCEO and IOF) summarized evidence from systematic reviews and meta-analyses (138). Adequate dietary protein is essential for optimal bone growth and the maintenance of healthy bone. In older individuals with osteoporosis, higher protein intake (≥0.8 g/kg body weight/day, above the current RDA) is associated with higher bone mineral density (BMD). It is also linked to a slower rate of bone loss and a reduced risk of hip fracture. Adequate calcium intake is necessary. The consensus noted no evidence that dietary protein, whether from animal or vegetable sources, is harmful to bone health.

Regarding drug interactions, both BCAAs and aromatic amino acids utilize the same LAT1 large neutral amino acid transporter across the BBB (139). High-dose BCAA supplementation could potentially compete for this transporter, reducing the amount of tyrosine and phenylalanine entering the brain and affecting catecholamine synthesis in Parkinson’s patients on levodopa. This possible interference is worth noting. Furthermore, BCAAs act as a major stimulator of mTORC1 signaling (130), suggesting potential interactions between these amino acids and drugs used to inhibit mTOR.

### 8.2 Bioavailability enhancement strategies

#### 8.2.1 Food processing technologies

Fermentation technology can be utilized to enhance the bioavailability of plant protein. Solid-state fermented plant food products (tempeh, sufu, and natto) significantly improve the protein digestibility of plants (140). Fungal and bacterial fermentations eliminate antinutrients and generate additional EAAs. These fermentations also impart distinctive texture and taste properties, including umami and kokumi compounds.

Germination technology enhances protein quality by activating endogenous enzyme systems. During germination, endogenous enzymes like protease, amylase, and phytase are activated (141), which increases the free amino acid content and degrades antinutrient factors (phytate, tannins), improving protein digestibility and mineral bioavailability. The combination of enzymatic treatments and fermentation may further increase the digestive absorption rate of plant protein. The combination of alcalase treatment and lactic acid bacteria (*Lactiplantibacillus plantarum* and *Levilactobacillus brevis*) fermentation has been tested (142), which increased the *in vitro* digestibility of pea protein by as much as 22.50% at 15 min and increased bioavailability by 38.40% according to a Caco-2 cell monolayer model; the average molecular weight decreased by 98.79%.

#### 8.2.2 Food pairing optimization

Cereal–legume pairing is a classic strategy for achieving protein complementation. Protein-rich plant foods such as traditional legumes, nuts, and seeds are sufficient to achieve full protein adequacy in adults consuming vegetarian or vegan diets (143). Concerns regarding amino acid deficiency in vegetarians have been substantially overstated. Cereal proteins are low in lysine but adequate in methionine, while legume proteins show the opposite pattern. Appropriate pairing can optimize the overall amino acid profile.

Avoiding interference from antinutritional factors requires appropriate food processing. Phytate can chelate minerals, including zinc, iron, and calcium, reducing their bioavailability. Tannins can bind to proteins and inhibit digestive enzyme activity (144). Traditional processing methods such as soaking, cooking, germination, or fermentation can effectively reduce the content of antinutritional factors (141).

### 8.3 Practical application recommendations

#### 8.3.1 General population

Specific recommendations have been proposed in the position paper of the PROT-AGE Study Group (106). To help maintain or regain lean body mass and function, it is recommended that older people (>65 years) have a daily protein intake of at least 1.0–1.2 g/kg body weight, with plant protein making up an adequate share of total protein consumption. Preference should be given to legumes (soybeans, peas, chickpeas), whole grains (quinoa, oats), and nuts and seeds (143). In terms of food intake, the ISSN Position Stand makes recommendations (145). An intake of 20 to 40 g of high-quality protein in each meal should be sufficient to maximally activate MPS. A common recommendation is a dosage of 0.25 g per kg body weight or an absolute dosage of 20–40 g of high-quality protein. The recommended intake of plant-derived branched-chain amino acids (BCAAs) for specific populations is summarized in Table 6.

**Table 6:** Recommended intake of plant-derived BCAAs for different populations.

Population category	Total protein recommendation (g/kg BW/day)	Plant protein proportion	BCAA source suggestions	Special considerations
Healthy adults	0.8–1.2 (103, 104)	≥50% (94, 95, 143)	Soy, pea, cereals, nuts (18, 139)	Emphasize protein complementation (18, 139)
Athletes/fitness enthusiasts	1.4–2.0 (141)	30–50% (116)	Soy protein isolate, pea protein (116, 117)	Timely supplementation after training (141)
Older adults (≥65 years)	1.0–1.5 (103, 104)	40–60% (139, 143)	Fermented soy products, mixed plant proteins (136, 137, 139)	Ensure adequate leucine intake (≥2–3 g/meal) (71, 103)
Metabolic syndrome patients	0.8–1.2 (103, 104)	≥60% (110, 143)	Legumes, nuts, whole grains (110, 123)	Limit red meat intake (102, 123, 143)
Vegetarians	1.0–1.2 (139)	100%	Diversified combinations (legumes + cereals + nuts) (18, 139)	Monitor serum amino acid levels (139)

#### 8.3.2 Special populations

##### 8.3.2.1 Athletes and fitness enthusiasts

Athletes and fitness enthusiasts have increased protein requirements. The ISSN Position Stand recommends a protein intake of 1.4–2.0 g/kg body weight/day for exercising individuals (145). This supports metabolic adaptation, repair, remodeling, and protein turnover. The position stand also notes that rapidly digested proteins rich in essential amino acids (particularly leucine) are most effective at stimulating muscle protein synthesis. A systematic review (including 19 randomized controlled trials) demonstrated that soy protein supplementation showed comparable effects to whey protein (120). These effects included muscle adaptations, metabolic and antioxidant status, hormonal response, and exercise performance, supporting soy protein as an effective plant protein alternative for athletic populations.

##### 8.3.2.2 Older adults (Sarcopenia prevention)

Older adults need more protein. The PROT-AGE Study Group recommends that healthy older adults (>65 years) consume at least 1.0–1.2 g protein/kg body weight/day (106), with a higher intake of 1.2–1.5 g/kg body weight/day recommended for those suffering from an acute or chronic illness. The European working group on sarcopenia in older people (EWGSOP2) 2019 updated consensus highlights that sarcopenia is a muscle disease (i.e., muscle failure) (146). Low muscle strength can be assessed through early screening with grip strength testing, chair stand testing, etc.

##### 8.3.2.3 Patients with metabolic syndrome

Patients with metabolic syndrome should prioritize high-quality protein sources. A prospective cohort study (including 131,342 participants and 36,115 deaths during follow-up) demonstrated important findings (147). After adjusting for major lifestyle and dietary risk factors, plant protein intake was associated with lower all-cause mortality (HR = 0.90, 95% CI: 0.86–0.95, per 10% energy increment). Among participants with at least one unhealthy lifestyle factor, animal protein intake was associated with higher cardiovascular disease mortality. Substituting plant protein for processed red meat protein was associated with a lower mortality risk.

##### 8.3.2.4 Vegetarians

Vegetarians need particular attention to protein diversity. Through appropriate combinations of traditional legumes, nuts, and seeds, adult vegetarians can fully meet their protein and essential amino acid requirements (143). The situation for older vegetarians is more complex, requiring greater attention to protein intake adequacy and quality.

#### 8.3.3 Clinical nutrition

Medical nutrition therapy regimens should be individualized according to disease type and metabolic status.

##### 8.3.3.1 Patients with liver cirrhosis and hepatic encephalopathy

A Cochrane systematic review (including 16 randomized controlled trials with 827 participants) demonstrated specific findings (148). Oral BCAAs improved clinical manifestations of hepatic encephalopathy (RR = 0.73, 95% CI: 0.61–0.88). High-quality evidence supports this conclusion. However, BCAAs had no significant effect on mortality (RR = 0.88, 95% CI: 0.69–1.11). Subgroup analysis showed that oral BCAAs (but not intravenous) had beneficial effects on overt hepatic encephalopathy. This systematic review supports the use of oral BCAAs as adjunctive therapy for hepatic encephalopathy.

##### 8.3.3.2 Liver cirrhosis patients with sarcopenia

A systematic review and meta-analysis demonstrated that the administration of BCAAs positively affects the parameters used to assess sarcopenia in patients with liver cirrhosis (149). The improvements include anthropometric indicators like mid-arm muscle circumference.

Monitoring indices should include muscle mass assessment (dual-energy X-ray absorptiometry or bioelectrical impedance analysis), grip strength measurement, gait speed (such as the Short Physical Performance Battery, Timed Up and Go test, or 400-meter walk test), and functional and nutritional status indicators including serum albumin and prealbumin levels (146).

## 9 Future perspectives and research directions

### 9.1 Basic research needs

Branched-chain plant-based amino acids: basic science progresses from the whole to the parts. More work is required to determine the individual roles of each BCAA. Dietary restriction of isoleucine alters hepatic and adipose metabolism (44). It enhances hepatic insulin sensitivity and ketogenesis and promotes energy expenditure through the FGF21-UCP1 pathway. Valine restriction also has similar but lesser effects. Leucine restriction does not produce these same effects. These findings challenge the conventional wisdom of treating the three BCAAs as functionally equivalent. New signaling pathways are still being discovered; for example, gut microbes encoded with BCAA metabolism-related genes could affect host blood sugar levels by modulating peripheral serotonin synthesis (150). Epigenetic mechanisms will be a new hotspot of research in future studies. Posttranslational modifications on histones affect chromatin conformation and transcriptional activity (151), which might have tissue specificity. The tissue-specific expression of these BCAA metabolic enzymes and how they are affected by diseases needs to be studied systematically (68).

A particular translational challenge concerns the highly publicized preclinical findings on isoleucine restriction and lifespan extension (103, 104). While these rodent studies are scientifically compelling, the mechanistic pathways involved (FGF21-UCP1 axis, sex-specific hepatic reprogramming, effects on tumor incidence) have not been characterized in human subjects. Furthermore, the degree of isoleucine restriction studied in mice (67% reduction) cannot be equated with the modest reduction in isoleucine intake that results from increasing plant protein consumption in human diets, given that even the lowest-isoleucine plant proteins (e.g., hemp, oat) still provide isoleucine above minimum requirements. Dedicated human dose–response studies, beginning with pharmacokinetic characterization of postprandial isoleucine kinetics under varying plant protein dietary conditions, are a prerequisite before isoleucine restriction can be considered a translatable clinical strategy for metabolic syndrome or aging.

### 9.2 Translational research directions

#### 9.2.1 Precision nutrition

The recommendation for personalization according to genotype has become one of the hot topics in research today. There are three levels for achieving precision nutrition (152). One level is dietary guidance based on population guidelines. Another level is personalized nutrition based on phenotype and lab test results. Finally, there is the third level: genotype-driven nutritional intervention. Polymorphisms of the genes involved in this pathway, such as BCKDH, may also impact how efficiently BCAAs are catabolized (153). Thus, there is potential at a genetic level to tailor individualized support. Another important direction is gut microbiota phenotype stratification. Different gut microbial compositions could modulate intestinal BCAA metabolism and host bioavailability (154). These are closely related to obesity and insulin resistance; large-scale clinical trials like PREDICT have validated some of these benefits (153). Personalized nutrition may have a greater impact on weight loss, glucose regulation, and diet compliance.

#### 9.2.2 Functional food development

The development of plant protein functional foods is progressing rapidly. Plant breeding for high-BCAA plants increases the amount and ratio of amino acids in legumes or cereals (155). This occurs through genetic improvement. New plant-based proteins are constantly being developed, incorporating new processing methods and utilizing various protein-containing materials such as soy, pea, and chickpea (156). Significant progress has also been made in the development of targeted delivery systems. Nanocarrier systems can protect bioactive peptides from gastrointestinal degradation (157). They utilize liposomes and polymer nanoparticles to improve bioavailability. Food protein nanoparticles show broad prospects in oral delivery systems (158), due to their biosafety and cost-effectiveness.

### 9.3 Clinical research needs

The most important deficiency in present clinical evidence is a lack of large sample long-term RCTs; most available RCTs had an intervention period ranging from weeks to 24 weeks (158), making it hard to assess the long-term impact on chronic disease hard endpoints. In future research directions, researchers are advised to conduct trials that include longer time points, incorporating more clinical outcomes (e.g., cardiovascular events or diabetes complications) into their study designs. Real-life data may compensate for the low generalizability of RCTs. Testing the effects of plant-based BCAA on different populations with a complicated background of medications would increase the generalizability of results. Geographic and ethnic variations should be addressed as well. The baseline diets and metabolic profiles are very different between Asian and Western populations (159). Cost-effectiveness analysis would yield the economic evidence that public health decision-makers need.

### 9.4 Public health significance

Plant-derived BCAA research has multiple public health values. Research findings can promote dietary guideline updates. Currently, only 45% of national dietary guidelines address environmental sustainability (160). Most do not distinguish between plant and animal protein health effects. Plant-based dietary patterns can simultaneously improve human health and reduce environmental burden (161). The construction of sustainable food systems is an international problem. According to the “planetary health diet,” developed by the EAT-Lancet Commission, certain changes are required by 2050 (162). Consumption of red meat should decrease by more than half. Consumption of nuts, fruits, vegetables, and legumes needs to be increased by over 100%, which would not only benefit human health but also decrease GHG emissions as well as the demand for irrigation water in agriculture (163).

### 9.5 Technological innovation directions

Technological advances will shape future developments in plant-derived BCAA research. Synthetic biology is an approach that allows the efficient manufacture of selected amino acids through microbial fermentation. Nanotechnology also has high potential to improve bioavailability (164) since nano-carriers protect active ingredients against degradation while allowing controlled delivery. The technology for smart nutrition sensing and monitoring is developing rapidly. Wearable devices and metabolomics sensors could monitor nutritional status in real time. An AI-assisted personalized nutrition plan would be one of the most promising directions; machine learning algorithms could integrate an individual’s genetic, metabolomic, and lifestyle data (165). They provide accurate diet recommendations. The research on AI-driven personalized diet interventions is growing fast. Deep learning and generative models allow for better nutrition advice (166).

## 10 Conclusion

In this review, we systematically examined the role of plant-derived BCAAs in regulating chronic inflammation and improving metabolic syndrome. Plant-derived BCAAs have sufficient sources and good bioavailability. Legumes like soybeans, peas, and lentils, as well as whole grain foods (e.g., quinoa, oats), are key contributors. Plant proteins are not always easy to digest, and the rate of release of their amino acids is sometimes low; therefore, digestion and hydrolysis processes (fermentation or germination) may be required to obtain higher quality proteins through enzymatic hydrolysis (156). Preclinical evidence from cell-based and rodent studies indicates that plant-derived BCAAs engage multiple anti-inflammatory pathways, including modulation of mTORC1 signaling, suppression of NF-κB activity, potential Nrf2-mediated antioxidant effects, and favorable gut microbiota modulation. Direct human evidence for several of these specific mechanistic pathways remains limited, and their contribution to the clinically observed benefits of plant-based diets cannot be established from mechanistic data alone. Clinical and epidemiological evidence supports associations between higher plant protein intake and improvements in individual MetS components, though the causal contribution of BCAAs specifically, as distinct from the broader plant food matrix, has not been isolated in human interventional studies. Replacing animal with plant proteins reduces CVD mortality according to epidemiological data. It is also linked to a lower incidence of type 2 diabetes. RCT data indicate that plant-based diets are capable of improving glycemic control, fasting insulin, and HOMA-IR (167). BCAA sources derived from plants could be more beneficial for human health than those derived from animals because of general properties of plant proteins, such as low levels of saturated fat, high fiber, and polyphenol antioxidant content, along with beneficial modulatory effects on gut microflora (161).

This research will have certain theoretical significance and practical application value. On the one hand, theoretically, this paper can supplement the scientific basis for plant-based diets by clarifying the specific biological effects and molecular mechanisms of plant-source BCAAs, which differ from those of animals. On the other hand, in terms of practical applications, it offers new options for the nutritional prevention and therapy of metabolic syndrome, facilitating the development of sustainable healthy nutrition styles. The advent of precision nutrition makes individualized plant protein interventions possible. Multi-dimensional approaches integrating genotype, gut microbiota phenotype, and metabolomics data will drive important changes (153). They will shift interventions from population-based to individualized approaches.

Objectively assessing current evidence reveals specific patterns. A critical appraisal of the existing evidence highlights an asymmetry between mechanistic depth and clinical validation. The mechanistic research base is relatively well-developed, with multiple signaling pathways characterized at the molecular level predominantly in cell culture and animal models. In contrast, direct human clinical evidence specifically attributing metabolic benefits to plant-derived BCAAs—as opposed to the totality of plant-based dietary patterns—remains limited, heterogeneous, and largely short-term. Mechanistic plausibility, however well-established in preclinical systems, should not substitute for clinical validation in informing dietary recommendations. Future research must prioritize the translation of preclinical mechanistic hypotheses into rigorously designed human interventional studies before plant-derived BCAA-specific recommendations can be made with high confidence. The long-term effects and optimal dosages need further study. The majority of RCTs have short intervention periods. Their impact in terms of hard endpoints (such as complications and cardiovascular events) requires confirmation. An individualized application strategy needs to be developed. Genetic variation, gut microbiota differences, and metabolic phenotypes influence responses to interventions, which must be incorporated into the design of future studies (153).

Plant-based BCAAs have great potential to control chronic inflammation and improve metabolic syndrome. In light of precision nutrition and sustainable diet concepts, in-depth studies on plant-derived BCAA health effects are both scientifically and socially significant, focusing on three core points: mechanism clarification, clinical verification, and application. This will foster the development of functional foods and the translation of science into dietary recommendations.

Despite the compelling mechanistic and epidemiological evidence reviewed herein, several important technical limitations must be acknowledged. First, BCAA quantification methodology remains inconsistent across studies. The use of targeted metabolomics (LC–MS/MS) versus enzymatic assays or HPLC introduces substantial inter-study variability in circulating BCAA measurements, complicating direct comparisons between cohorts (129). Standardized, validated analytical protocols for both dietary BCAA assessment and plasma quantification are urgently needed. Second, dietary assessment instruments used in observational studies—including 24-h dietary recalls and food frequency questionnaires—have inherent limitations in capturing true BCAA intake, particularly given the substantial variation in BCAA content arising from food variety, growing conditions, and culinary processing (18). Third, the overwhelming reliance on short-term RCTs (median duration <24 weeks) precludes conclusions regarding the long-term efficacy and safety of plant-derived BCAA interventions on hard clinical endpoints such as incident cardiovascular events, diabetes complications, and all-cause mortality (124, 125). Fourth, animal-to-human translation presents persistent challenges: the metabolic effects of isoleucine restriction demonstrated in rodent models (102, 103), while highly promising, may not directly recapitulate human physiology given fundamental differences in BCAA metabolic flux rates, gut microbiota composition, and endocrine regulation. Fifth, most intervention studies have been conducted in homogeneous populations (predominantly Western, middle-aged, and relatively healthy), severely limiting the generalizability of findings to diverse ethnic groups, older adults with multimorbidity, and populations in low- and middle-income countries where plant-based diets are the default rather than a deliberate choice.

Reflections on Future Research Priorities.

Moving forward, the field requires a fundamental shift in the research paradigm rather than merely incremental additions to existing evidence. The prevailing reductionist approach—isolating individual BCAAs or single plant protein sources—must give way to systems-level investigations that capture the complexity of whole dietary patterns, food matrices, and their interactions with host biology. Specifically, future research should prioritize: (i) Multi-omics integration: the concurrent profiling of genomics (BCKDH polymorphisms), gut metagenomics, plasma metabolomics, and proteomics within the same individuals will be essential for identifying BCAA-responsive subpopulations and mechanistic subtypes of MetS (153); (ii) Precision nutrition trial design: adaptive, biomarker-stratified RCTs that pre-select participants based on BCAA metabolic phenotype (e.g., BCAA catabolic efficiency, gut microbiota composition) are needed to detect treatment effects that may be diluted in heterogeneous populations (152); (iii) Head-to-head comparison of specific plant protein sources: rather than treating “plant protein” as a monolithic category, rigorous comparative trials should distinguish between legume-, cereal-, and microalgae-derived protein interventions, given their distinct BCAA profiles, accompanying phytochemicals, and gut microbiota-modulating capacities (18, 24); (iv) Long-duration pragmatic trials: embedding BCAA-focused dietary interventions within real-world healthcare settings, leveraging electronic health records and remote dietary monitoring technologies, will provide more ecologically valid evidence than tightly controlled laboratory studies; (v) Sex- and age-stratified analyses: the sexually dimorphic metabolic effects of isoleucine restriction (102, 103) and the age-dependent anabolic resistance to plant protein (43) underscore the necessity of stratified analyses in future trials rather than pooled reporting. Translating the mechanistic depth already achieved in preclinical research into actionable, population-specific dietary recommendations for MetS prevention and management remains the central challenge—and the central opportunity—for this rapidly evolving field.
